# A Smartphone-Based Self-management Intervention for Bipolar Disorder (LiveWell): User-Centered Development Approach

**DOI:** 10.2196/20424

**Published:** 2021-04-12

**Authors:** Geneva K Jonathan, Cynthia A Dopke, Tania Michaels, Andrew Bank, Clair R Martin, Krina Adhikari, Rachel L Krakauer, Chloe Ryan, Alyssa McBride, Pamela Babington, Ella Frauenhofer, Jamilah Silver, Courtney Capra, Melanie Simon, Mark Begale, David C Mohr, Evan H Goulding

**Affiliations:** 1 Department of Psychiatry and Behavioral Sciences Feinberg School of Medicine Northwestern University Chicago, IL United States; 2 Pediatrics Loma Linda Children’s Hospital Loma Linda, CA United States; 3 Department of Psychology University of Regina Regina, SK Canada; 4 Department of Social Work UPMC Western Psychiatric Hospital Pittsburgh, PA United States; 5 Department of Psychology Stony Brook University Stony Brook, NY United States; 6 Department of Psychology School of Science and Engineering Tulane University New Orleans, LA United States; 7 Vibrent Health Fairfax, VA United States

**Keywords:** behavioral intervention technology, mHealth, bipolar disorder, depression, illness management, smartphone, behavior change, early warning signs, self-management, qualitative

## Abstract

**Background:**

Bipolar disorder is a serious mental illness that results in significant morbidity and mortality. Pharmacotherapy is the primary treatment for bipolar disorder; however, adjunctive psychotherapy can help individuals use self-management strategies to improve outcomes. Yet access to this therapy is limited. Smartphones and other technologies have the potential to increase access to therapeutic strategies that enhance self-management while simultaneously providing real-time user feedback and provider alerts to augment care.

**Objective:**

This paper describes the user-centered development of LiveWell, a smartphone-based self-management intervention for bipolar disorder, to contribute to and support the ongoing improvement and dissemination of technology-based mental health interventions.

**Methods:**

Individuals with bipolar disorder first participated in a field trial of a simple smartphone app for self-monitoring of behavioral targets. To develop a complete technology-based intervention for bipolar disorder, this field trial was followed by design sessions, usability testing, and a pilot study of a smartphone-based self-management intervention for bipolar disorder. Throughout all phases of development, intervention revisions were made based on user feedback.

**Results:**

The core of the LiveWell intervention consists of a daily self-monitoring tool, the Daily Check-in. This self-monitoring tool underwent multiple revisions during the user-centered development process. Daily Check-in mood and thought rating scales were collapsed into a single wellness rating scale to accommodate user development of personalized scale anchors. These anchors are meant to assist users in identifying early warning signs and symptoms of impending episodes to take action based on personalized plans. When users identified personal anchors for the wellness scale, the anchors most commonly reflected behavioral signs and symptoms (40%), followed by cognitive (25%), mood (15%), physical (10%), and motivational (7%) signs and symptoms. Changes to the Daily Check-in were also made to help users distinguish between getting adequate sleep and keeping a regular routine. At the end of the pilot study, users reported that the Daily Check-in made them more aware of early warning signs and symptoms and how much they were sleeping. Users also reported that they liked personalizing their anchors and plans and felt this process was useful. Users experienced some difficulties with developing, tracking, and achieving target goals. Users also did not consistently follow up with app recommendations to contact providers when Daily Check-in data suggested they needed additional assistance. As a result, the human support roles for the technology were expanded beyond app use support to include support for self-management and clinical care communication. The development of these human support roles was aided by feedback on the technology's usability from the users and the coaches who provided the human support.

**Conclusions:**

User input guided the development of intervention content, technology, and coaching support for LiveWell. Users valued the provision of monitoring tools and the ability to personalize plans for staying well, supporting the role of monitoring and personalization as important features of digital mental health technologies. Users also valued human support of the technology in the form of a coach, and user difficulties with aspects of self-management and care-provider communication led to an expansion of the coach's support roles. Obtaining feedback from both users and coaches played an important role in the development of both the LiveWell technology and human support. Attention to all stakeholders involved in the use of mental health technologies is essential for optimizing intervention development.

## Introduction

Bipolar disorder is characterized by recurrent episodes of mania, hypomania, depression, and mixed states. Many individuals experience multiple acute episodes, long episode durations, and interepisode symptoms [1-5]. As a result, individuals with bipolar disorder are symptomatically ill about half of the time, and three-quarters of those affected never achieve full recovery of psychosocial function [1,6]. Pharmacological management is the primary treatment for bipolar disorder and effectively reduces relapse risk and symptom burden [7,8]. The addition of empirically supported psychotherapy to pharmacotherapy can further lower relapse rates, decrease symptoms, and improve quality of life [9-17].

Although psychotherapy delivered during research trials has proven helpful, only about half of individuals with bipolar disorder receive any therapy [18,19]. Enhancing access to content and tools from empirically supported psychotherapy is essential to improving treatment for bipolar disorder. Digital mental health technologies, including web and smartphone-based applications, provide a means to increase the availability of empirically supported psychotherapeutic strategies for managing mental health problems [20-24]. In addition to access barriers, current tools for bipolar disorder self-management can be improved [14,25-28]. Relative to face-to-face treatment, mental health technologies can advance intervention functionality by providing real-time assessments, feedback, and provider alerts [29]. Many people with bipolar disorder are interested in utilizing self-management strategies to stay well, and strategies used by individuals who are doing well overlap significantly with the content of empirically supported psychotherapies for bipolar disorder [30-33]. This overlap suggests that smartphone-based interventions delivering self-management strategies derived from empirically supported psychotherapies may meet user needs and support user engagement [34-37].

To address the need for increased access to and enhancement of empirically supported therapy for bipolar disorder, we designed and developed LiveWell, a smartphone-based self-management intervention (Clinicaltrials.gov NCT02405117, NCT03088462). Description of intervention development is essential to support ongoing improvement and dissemination of technology-based mental health interventions [38-42], and thus, this paper describes the user-centered development of LiveWell. The development approach aims to ground LiveWell in the lived experiences of individuals with bipolar disorder to create an intervention that encourages the development and long-term use of self-management strategies for living well with bipolar disorder [43-45]. The user-centered development was carried out in phases, consisting of (1) an initial 12-week field trial of a simple smartphone app for self-monitoring of behavioral targets, (2) design interviews and usability testing of a self-management app, and (3) an 8-week pilot trial of the complete LiveWell intervention, including both technology and human support.

## Methods

### Users

The study was reviewed and approved by Northwestern University’s institutional review board. Users were recruited via fliers placed at university-affiliated and private outpatient mental health practices. Eligible users were 18-65 years old and had a DSM-IV (Diagnostic and Statistical Manual of Mental Disorders–fourth version) diagnosis of bipolar disorder with a minimum of 2 acute mood episodes within 2 years of enrollment. Individuals were excluded if they (1) were not in current psychiatric care; (2) met criteria for a substance-use disorder within the last 6 months; (3) met criteria for another psychiatric diagnosis, or had symptoms for which participation in the study was either inappropriate or dangerous (including current severe suicidal ideation or a serious suicide attempt in the last 12 months); (4) were pregnant or planned to become pregnant; (5) had a visual, hearing, voice, or motor impairment that would prevent completion of the study procedures or limit smartphone use; or (6) were unable to speak or read English. As the intervention primarily targets mood episode relapse prevention, a current mood episode at the baseline assessment was an additional exclusion criterion for the pilot study.

Before an initial telephone screening, users completed informed consent by telephone or online. Users completed written consent before a face-to-face (F2F) clinic visit. Initial screening to establish a bipolar disorder diagnosis was conducted via telephone using the Mini–International Neuropsychiatric Interview [46]. If eligible, users completed an F2F interview with a study clinician (psychiatrist or psychologist) using an abbreviated version of the Affective Disorders Evaluation and the Clinical Monitoring Form [47,48]. Individuals with a confirmed diagnosis at the clinic visit enrolled in the field trial, design interview, and/or usability testing and could additionally continue to the next phase if they chose. For the pilot study, users also completed a baseline telephone assessment using the Clinical Monitoring Form to assess current clinical status. All individuals who participated in earlier phases of intervention development were offered an opportunity to participate in subsequent development phases. Users were compensated for their time and travel costs: US $10 was given toward travel costs and the telephone assessment; US $15 was given for participation in the clinical assessment, baseline and monthly telephone assessment, exit interview, and app training; and US $25 was given for the design and usability interviews.

### Procedures

#### Field Trial

A 12-week field trial was completed by 4 users to assess a simple self-monitoring smartphone app. The research team collaborated with the Center for Behavioral Intervention Technologies (CBITS) at Northwestern University to develop an Android app with self-report data collection, encryption, and transmission to a secure server. Users were provided with a smartphone with a data plan and had an F2F meeting with a study staff member (coach) who used a structured script and handouts to introduce the app (Multimedia Appendix 1). The coach used structured interview scripts to gather feedback about the training after the session and app use after the field trial (Multimedia Appendix 1).

#### Design Sessions and Usability Testing

Design sessions were conducted with 4 users from the initial study and 1 additional user, for which a structured script and handouts (Multimedia Appendix 2) were used to simulate an F2F app training session with a coach. Users were provided with a smartphone app mock-up (Multimedia Appendix 3), asked to imagine using the app for the next 16 weeks, and instructed to think and ask any questions out loud. The research team collaborated with CBITS to extend the self-monitoring Android app to include information and tools to help users engage in bipolar disorder self-management. The 5 users from the design sessions attended F2F usability testing sessions that employed structured scripts and scenarios (Multimedia Appendix 4). Users were given 5-10 minutes to explore the app and asked to share their general impressions. Then, users read one assigned lesson and another of their choice and were asked to provide feedback about the lesson's usefulness, length, and coverage. Next, users read aloud scenarios that mimicked real-life situations: medication nonadherence, sleeping too little, and experiencing early warning signs of depression. After reading each scenario, users completed a daily check-in based on the scenario and received automated feedback from the app based on their self-report data. Users were asked to discuss the usefulness of this feedback and how it could be improved. All users were given a posttask questionnaire to rate the overall usability of the app (Multimedia Appendix 5).

#### Pilot Study

To test the complete intervention, 11 users, including 4 who attended the usability testing, completed an 8-week pilot study. Users were provided with a smartphone and a data plan. They had an F2F meeting with a coach who used a structured script and handouts (Multimedia Appendix 6) to instruct them on the use of the app and the coach's role. Following this meeting, users completed 6 phone calls (weeks 1-4, 6, and 8) with a coach; the coach used structured scripts (Multimedia Appendix 6) to support app use adherence, self-management strategy use (including the development of personalized wellness plans; Multimedia Appendix 7), and communication with clinical care providers. After completing the pilot, users completed a structured exit interview (Multimedia Appendix 8) and an exit questionnaire (Multimedia Appendix 9) to provide feedback about the app’s usability.

### Analysis

#### Instant Data Analysis

The research team utilized an instant data analysis approach across all development phases to make iterative changes to the technology and coaching based on user feedback [49]. This approach reduces the time needed for analysis while also identifying usability issues [50,51]. Immediately after observing, interacting with, or receiving feedback from users, study staff wrote memos documenting users' problems or comments. The design team (coaches, programmers, the project manager, and team leaders) discussed these memos until they achieved consensus on necessary changes [52]. These discussions sometimes prompted a return to the literature to provide additional information to make design decisions. During the pilot study, coaches' experiences with the technology and the users were increasingly incorporated into the design discussions and decisions. Based on the number of users, the field trial and usability studies (n=4-5) should identify 55%-85% of problems with app usability, and the pilot study (n=11) should detect 80%-95% of usability problems [53-56]. In addition to the traditionally defined users, 4 coaches and 1 team leader provided human support during different stages of the development process.

#### Analysis of Usability Testing Posttask and Pilot Study Exit Questionnaires

Participant responses to the usability testing posttask questionnaire (n=5) and pilot study exit questionnaire (n=11) were used to assess usability (Multimedia Appendices 5 and 9). To provide summary assessments, responses from the 7-point response scales were collapsed into 2 categories: disagree/strongly disagree and agree/strongly agree.

#### Analysis of Pilot Study Exit Interviews

The exit interviews (n=11) were transcribed verbatim and used for thematic analysis [57]. Then, 3 researchers independently conducted a preliminary round of coding during which transcripts were partitioned into excerpts (transcript lines conveying a codable unit) and exported to Microsoft Excel spreadsheets (Multimedia Appendix 10). App usability subthemes were inductively coded and deductively grouped into larger themes. Coders used nominal group consensus, meeting with a moderator to discuss, clarify differences in, and finalize codes [52].

#### Analysis of Rating Scale Anchors

Thematic analysis was used to investigate users' personalized anchors for the mood and thought rating scales used during the field trial (n=4) and the wellness rating scales used during the design sessions (n=5) and pilot study (n = 11) [57]. Anchors were entered into Excel spreadsheets (Multimedia Appendix 11), and 2 researchers inductively coded subtypes. A third researcher reviewed the codes, and a consensus process was used to finalize anchor subtypes [52]. Anchor subtypes were then deductively grouped into broader categories (anchor types) based on a literature review describing early warning signs experienced by individuals with bipolar disorder prior to an episode [11,12]. The research team discussed the overall coding scheme and developed definitions and examples of the anchor types and subtypes. Then, 2 researchers who were not involved in the initial coding and development used these definitions and examples to code the anchor types and subtypes across all 3 scales; the joint probability of agreement was 90% for subtypes and 87% for types.

## Results

### Users

At study enrollment, 12 users were included and were 21-62 (mean 38, SD 14) years old. Of these 12 users, 4 were male and 8 were female; 12 were non-Hispanic white; 3 were married or living as married, 3 were divorced, and 6 were never married; 5 had completed some college, 2 had completed college, and 5 had completed more than college; 2 were students, 6 were employed, 2 were unemployed, and 2 were on disability.

### Intervention Overview

Similar to existing, empirically supported F2F psychotherapy interventions for bipolar disorder, the LiveWell intervention aims to decrease episode relapse, reduce symptom burden, and improve quality of life [3,4,10-15,58-60]. The intervention seeks to achieve these outcomes by assisting individuals with managing behaviors proposed to underlie the impact of existing therapies [7,11-13,15,18,19,31,61,62]. LiveWell thus emphasizes identifying early warning signs and symptoms of relapse, developing plans and monitoring for relapse, and then enacting and adjusting plans as needed. In addition, LiveWell engages users in a similar process to support taking medications as prescribed, obtaining adequate sleep duration, and maintaining regular routines. LiveWell also addresses strengthening social support, managing stressors, and engaging in healthy habits regarding diet, exercise, and substance use.

**Figure 1:**
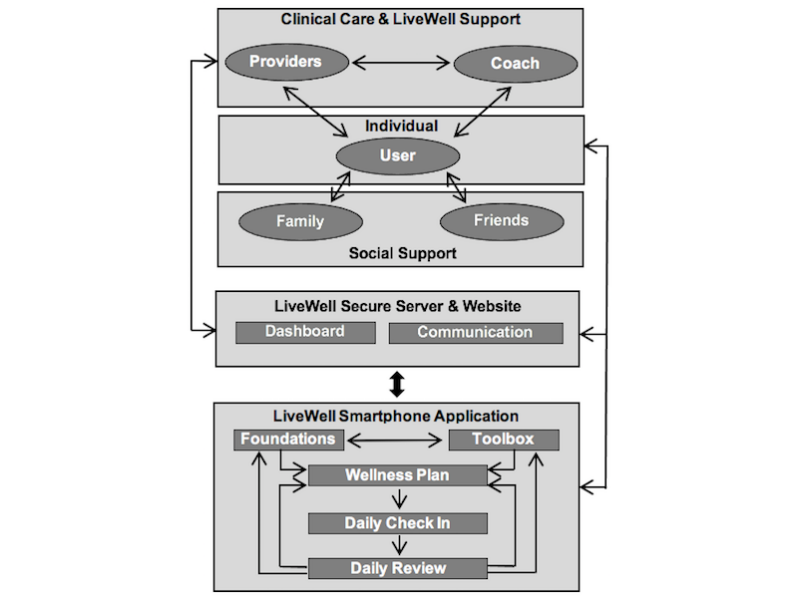
Intervention overview.

LiveWell consists of technological and human support components, including a smartphone app, a secure server, a website, and coaching support (Figure 1). The smartphone app consists of 5 components: Foundations, Toolbox, Wellness Plan, Daily Check-in, and Daily Review. It provides foundational information on bipolar disorder self-management (Foundations) and a toolbox with self-assessment surveys and skills practice (Toolbox). The Foundations and Toolbox components support developing a personalized Wellness Plan to reduce relapse risk and manage signs and symptoms. The core of the intervention is a Daily Check-in, in which users monitor medication adherence, sleep duration, routine, and wellness. Based on Daily Check-in data, the Daily Review provides tailored feedback that directs users to relevant psychoeducation content (eg, using lifestyle skills to reduce relapse risk or coping skills for managing early warning signs and symptoms).

LiveWell also utilizes human support, a coach to improve app use adherence, self-management, and communication with mental health providers [63]. The app provides data summaries and alerts via a secure server and website to help the coach provide support and facilitate communication with mental health care providers. Personnel without professional mental health training provide coaching support to reduce costs and increase access [64,65]. A clear division of labor between the technology and the coach ensures that coaches operate within the scope of nonclinical practice. The technology functions as the psychotherapeutic strategy expert that provides status summaries and alerts to the coach, who uses structured scripts and flowsheets to serve as a technology use concierge.

### Field Trial

Monitoring is a major determinant of behavior change [41] and an essential strategy of empirically supported bipolar disorder psychotherapies [3,11,25,60]. Individuals with bipolar disorder are interested in using self-monitoring tools [30,31]. Thus, the initial development of LiveWell focused on creating a smartphone-based self-monitoring tool for tracking moods, thoughts, sleep, and routine. In particular, the ability to distinguish between different levels of wellness (eg, doing well or responding as expected to events versus experiencing early warning signs of an episode) may be essential to staying well [33].

**Figure 2:**
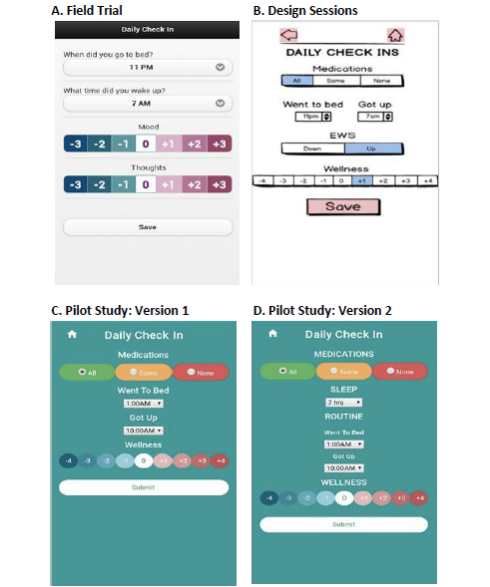
Daily Check-in development. (A) Field trial; (B) Design sessions; (C) Pilot study, version 1; (D) Pilot study, version 2.

Due to the potential importance of this ability, the field-trial Daily Check-in included 7-point scales to monitor mood and thoughts (Figure 2, Table 1). During the app training session, coaches helped users establish mood and thought anchors (ie, words describing their prior experiences at different wellness levels) to make these scales more personally relevant and useful. However, coaches noticed that users' mood and thought anchors often overlapped (eg, “planning for my future” as a mood anchor; Table 1), and some anchors appeared to reflect behaviors, physical symptoms, and changes in motivation (eg, “sluggish” as a thought anchor; Table 1). In addition, users reported that the 7-point range was too restrictive (eg, “If people are more depressed, it would probably be useful to have a wider scale to describe it”). As a result, the mood and thought rating scales were collapsed into a single 9-point wellness rating scale (Figure 2).

Users were also given the option to complete the Daily Check-in multiple times a day, but coaches noted that allowing multiple check-ins did not appear to elicit the reflection needed to identify different wellness levels. Instead, the Daily Check-in seemed to be capturing momentary reactions to daily hassles and uplifts [66]. To encourage users to engage in reflective monitoring rather than in-the-moment rating, the Daily Check-in was restricted to allow only one check-in per day, and the coaching scripts were adjusted to encourage reflection on wellness status using the personalized anchors.

**Table 1:** Field trial: mood and thought rating scales (n=4).

Rating	Definition	Personalized anchor (user ID)	
		Mood anchor	Thought anchor
+3 Severe up	Most manic for you; extremely problematic	Ecstatic (2001) Uber social (2002) Sleep less than 12 hours (2008)	Racing (2001) Can work very fast (2002) Nonsensical (2003)
+2 Moderate up	Moderately problematic	Overly hopeful (2001) Full energizer (2008)	Feel up (2002) Have energy to study (2008)
+1 Slight up	Normal variation; explainable by recent or upcoming events	Anticipation (2001) Life is manageable (2002) Talkative (2003)	Realistic (2001) Feel competent (2002) Can study and enjoy it (2008)
0 Well	Doing well	Satisfied (2001) Cheerful (2003) Planning for my future (2008)	Reasonable (2001) Steady (2003) Learn new things (2008)
-1 Slight down	Normal variation; explainable by recent or upcoming events	Disappointed (2001) All or nothing kind of thinking (2002) Tired (2003)	Sluggish (2001) Normal thought rate (2003) Anxious (2003)
-2 Moderate down	Moderately problematic.	Cry easily (2002) Tired (2003).	Hopeless (2001) Don't feel like myself (2002)
-3 Severe down	Most depressed for you; extremely problematic	Despondent (2001) Emotional pain (2002) Overly lethargic (2003).	Despair (2001) Overly critical (2003) I can't study (2008)

### Design Sessions and Usability Testing

Content and tools for the app and coaching support were developed based on information from empirically supported psychotherapies for bipolar disorder [3,12,13,15], health psychology behavior change theories [39,40,67-74], and chronic disease self-management models [75-83]. Design sessions and usability testing were then conducted to obtain user feedback on the overall app design, Daily Check-in, Daily Review, Foundations lessons, and the F2F coaching app training session.

During the design sessions, the Daily Check-in had separate early warning sign buttons to emphasize the importance of early warning signs (Figure 2). User feedback indicated these buttons were unnecessary and confusing with the transition to the 9-point rating scale. Thus, the Daily Check-in was simplified by removing these buttons, and early warning signs were clearly defined as +2 and -2 on the wellness rating scale (Table 2). Additionally, the Foundations content and coaching scripts were updated to emphasize the use of the wellness rating scale for early warning signs recognition.

During usability testing, user feedback suggested that exercise and diet were not adequately addressed. Therefore, Foundations and Toolbox content was added to cover the importance of exercise and diet [84-86]. User feedback also suggested that finding the right doctor and establishing a therapeutic alliance was not adequately discussed and that the rationale and approach to identifying a hospital in the case of a severe episode were unclear. As a result, the team lesson about working with a psychiatrist was expanded, the rationale for learning about a hospital for inpatient care was clarified, and information about using mental health directives was added.

In their posttask questionnaire responses, users indicated that the app's overall design and organization were straightforward, easy to use and understand, reasonable in terms of time commitment, and met their expectations (Table 3, Multimedia Appendix 5). Users also indicated that both the Foundations and Daily Review were interesting, relevant, and taught them something new.

**Table 2:** Design sessions: wellness rating scale (n=5).

Rating	Definition	Personalized wellness anchor (user ID)
+4 Severe up	Poor judgment, dangerous behaviors, not sleeping, hallucinations/delusions	Spend too much money (2002) Not sleeping (2003) Breaking stuff (2005)
+3 Moderate up	Many symptoms day to day, manic episode probably happening, difficult to maintain activities/routine	Physically energized (2001) Everything amplified (2002) Cussing out strangers (2005)
+2 Mild up	Some ongoing symptoms or early warning signs; manic episode may be coming, can still maintain activities/routine	Mood more volatile (2002) Binge drinking (2005) Walking for a long distance (2008)
+1 Slight up	Response to recent/upcoming good event, likely normal variation in wellness, understandable and manageable	More hopeful (2002) Chipper (2005) Exercise a little bit (2008)
0 Balanced	Neither up nor down, doing well	Engaged in life (2002) Like being around people (2005) Mood happy (2008)
-1 Slight down	Response to recent/upcoming bad event, likely normal variation in wellness, understandable and manageable	I'd rather sit at home (2001) Slight upset (2003) Sarcastic (2005)
-2 Mild down	Some ongoing symptoms or early warning signs, depressive episode may be coming, can still maintain activities/routine	Angry (2002) Selectively returning messages (2005) Loss of energy level interferes with daily tasks (2008)
-3 Moderate down	Many symptoms day to day, depressive episode probably happening, difficult to maintain activities/routine	Using sleep to avoid life (2001) Skipping meals (2005) Slow thought process (2008)
-4 Severe down	Serious ideas about suicide, immobilized, dangerous behaviors, disrupted sleep, hallucinations/delusions	Lack of motivation to do things (2003) Suicide planning (2005) Loss of interest in everything (2008)

**Table 3:** Usability testing: posttask questionnaire (n=5).

Section and usability type			Question		DSD^a^		ASA^b^
**Overall**							
	Interface quality	This application is visually appealing.		0		80	
	Interface quality	It was easy to move from one page to another.		0		80	
	Ease of learning	The overall organization of the application is easy to understand.		0		100	
	Interface quality	Individual pages are well designed.		0		80	
	Ease of learning	Terminology used in this application is clear.		0		80	
	Satisfaction	The content of the application met my expectations.		0		60	
	Satisfaction	I would be likely to use this application in the future.		0		80	
	Ease of use	I was able to complete my tasks in a reasonable amount of time.		0		80	
	Ease of use	Overall, the application is easy to use.		0		100	
**Foundations**							
	Ease of learning	I found the lessons easy to understand.		0		80	
	Usefulness	I found the lessons interesting.		0		80	
	Usefulness	I found the lessons relevant to me.		0		60	
	Usefulness	I learned something new from the lessons.		0		60	
	Usefulness	I was motivated to make a change after reading the lessons.		0		60	
**Daily Review**							
	Ease of learning	I found the daily review easy to understand.		0		80	
	Usefulness	I found the daily review interesting.		0		80	
	Usefulness	I found the daily review relevant to me.		0		60	
	Usefulness	I learned something new from the daily review.		0		80	
	Usefulness	I was motivated to make a change after completing the daily review.		0		80	

^a^DSD: disagree/strongly disagree.

^b^ASA: agree/strongly agree.

### Pilot Study

The pilot study tested the complete intervention, including the technology and human support. At the F2F session, coaches helped users identify personalized wellness anchors (Table 2) and set mutually agreed-upon target goals (medication adherence, sleep duration, routine bedtime and rise time, and wellness rating range) for daily monitoring (Figure 1). The coaches encouraged users to set goals known to facilitate staying well [11-13,87], such as taking their medications 100% of the time, sleeping the recommended amount each day (7-9 hours, with 6-10 hours reasonable for some), going to bed and starting their day within a 1.5-hour window, and keeping their wellness ratings within a balanced range (-1/+1; Table 2).

**Figure 3:**
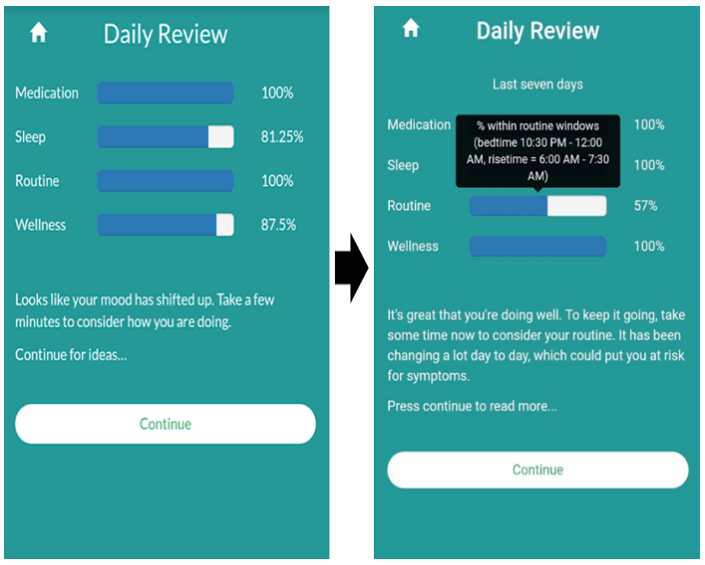
Daily Review development.

Users found differentiating between maintaining a regular routine and getting the right amount of sleep confusing. Therefore, a separate button for recording sleep duration was added to the Daily Check-in (Figure 1), and coaching scripts were updated to clarify the behavioral targets and goal setting process (Multimedia Appendix 6). Users also expressed difficulties understanding the Daily Review feedback regarding goal achievement. The first page of Daily Review feedback uses bar graphs to display the percent of days over the last 7 days that the user met their target goal (Figure 3). A title was added to the Daily Review bar graphs to indicate that the percentage bars correspond to goal achievement over the last 7 days. A hover bar feature was also added so that users could view their personalized goals for each behavioral target (Figure 3). Some users found the Daily Review feedback repetitive when target goals were consistently not met. To allow users to sample additional Daily Review content under these conditions, a Review Something Else component was added and made available after completing the suggested Daily Review feedback. Coaches found that users did not always follow up on Daily Review feedback to contact their providers. To address this, when feedback prompting communication with providers was given, a pop-up message with a link to one’s psychiatrist’s phone number was added to the Daily Review. Email alerts to coaches were also added, and coaching scripts were developed to help the coaches contact the user and psychiatrist under these conditions [63].

At week 4, users were asked to complete reading the Foundation lessons, and coaches assisted users in developing a lifestyle plan to reduce relapse risk (Table 4) and a coping plan for managing signs and symptoms (Table 5). Originally, generic suggestions for the plans were included. However, when coaches asked users to personalize their plans, some wanted to keep the generic plan. Generic plans were removed for the diet, exercise, substances (Attend), stress management (Tranquil), and build-support (Social) sections of the Reduce Risk plan to encourage users to think of relevant personal plans (Multimedia Appendix 7). Users' personalized goals for sleep duration and bedtime/rise time windows, and a generic recommendation to take all of their psychiatric medications daily, were retained. Coaching scripts were adapted to encourage the use of implementation intentions for planning [88-90], and users were given examples of implementation intentions in the generic risk-reduction plans (eg, “If you miss medications for more than 3-4 days in a row, then discuss with your psychiatrist”).

**Table 4:** Pilot study: lifestyle plan for reducing risk (n=11).

Target	Personalized plan (user ID)
Sleep	Keep routine. (2001) Sleep diary if not going well (2005) If having trouble sleeping, try sleep medication. (2066)
Medication	Talk to psychiatrist about current medication. (2003) Solve how to get around barriers or hurry/inattentiveness. (2005) Take every medication at the same time every day (2041)
Attend	Pay attention to sugar and processed foods. (2065) Do yoga/tai chi at least 3 times a week. (2066) Limit caffeine to 1 cup of coffee a day. (2086)
Routine	Don't skip meals. (2003) Exercise at a consistent time. (2008) If having trouble getting up, set an alarm. (2063)
Tranquil	Do a crossword puzzle. (2016) Listen to relaxation strategies. (2041) Don't over-schedule to avoid too much stimulation. (2066)
Social	Go to work with a positive attitude. (2001) Explore churches. (2066) Socialize with friends or family at least twice a week. (2086)

**Table 5:** Pilot study: coping plan for managing signs and symptoms (n=11).

Rating	Definition	Personalized anchor and plan (user ID)	
		Anchor	Plan
+4 Severe up	Poor judgment, dangerous behaviors, not sleeping, hallucinations/delusions	Super aggressive (2005) Invincible (2001) Risky activities (2041)	Increase supervision. (2063) Let supports know. (2086)
+3 Moderate up	Many symptoms day to day, manic episode probably happening, difficult to maintain activities/routine	Hardly sleeping (2001) Really angry (2005) Excessive energy (2008)	Stick to routine. (2016) Lean on family for support. (2008)
+2 Mild up	Some ongoing symptoms or early warning signs, manic episode may be coming, can still maintain activities/routine	Bizarre thoughts (2016) Short-tempered (2005) Sleep less (2001)	Be conscious, notice signs. (2003) Ask supports if noticing manic symptoms. (2005)
+1 Slight up	Response to recent/upcoming good event, likely normal variation in wellness, understandable and manageable	Friendly (2005) More social (2016) More active (2041)	Watch substance intake, especially alcohol, during the week. (2063) Moderate alcohol consumption. (2001)
0 Balanced	Neither up nor down, doing well	Balanced sleep (2016) Energy level is normal (2001) Productive (2001)	Recognize you are doing a great job! (2041) Eat healthy and exercise. (2063)
-1 Slight down	Response to recent/upcoming bad event, likely normal variation in wellness, understandable and manageable	Pessimistic reaction to events (2005) Can't motivate self (2016) Irritable (2003)	Let self feel sad, then move on because [I] can't change events. (2008) Do things that make you happy. (2003)
-2 Mild down	Some ongoing symptoms or early warning signs, depressive episode may be coming, can still maintain activities/routine	Crying (2016) Start losing appetite (2005) More restless (2008)	Try not to sleep too much or spend too much time in bed. (2003) Live day by day. (2008)
-3 Moderate down	Many symptoms day to day, depressive episode probably happening, difficult to maintain activities/routine	Trouble leaving house (2041) Oversleeping (2016) Grooming not shaving (2008)	Go to [my] mom's house. (2016) Call family supports. (2041)
-4 Severe down	Serious ideas about suicide. Immobilized. Dangerous behaviors. Disrupted sleep. Hallucinations/delusions.	Actively trying to harm self (2003) Odd thoughts (2008) Hate cycle (2016)	See doctor immediately. (2008) Contact those you trust. (2003)

Users reported confusion and difficulties with setting and achieving target goals and did not consistently follow up with Daily Review recommendations to contact providers; thus, the coach role was expanded from app use support [65] to more active support for self-management and clinical care communication. Clear coach email alerts and dashboard summaries, scripts, and flowsheets were developed to support these additional roles [63]. The coaching scripts were modified to emphasize creating target behavioral goals, assisting users with developing plans, reinforcing success, and making adjustments when goals were not met (Multimedia Appendix 6). This increased self-management support included developing flowsheets and tip sheets to help coaches guide the user to appropriate self-management content [63]. Questions from coaches about how to address worsening symptoms resulted in additional structured protocols to assess suicidality and functional impairment, including clear instructions on when to call for real-time clinical support. Details of the rationale and implementation of the coaching support are described elsewhere [63].

### Pilot Study Exit Analysis

When users finished the 8-week pilot study, they were asked to complete a usability questionnaire (Multimedia Appendix 9) [91,92]. Responses are summarized in Table 6 for questions in which ≥7/11 users rated an item as “strongly agree” or “agree,” or ≥3/11 users rated an item as “strongly disagree” or “disagree.” All users found the Daily Check-in easy to use, and most indicated that it made them more aware of their early warning signs and sleep duration. Users expressed that the Foundations were easy to understand, about the right length, and relevant. They reported that they liked being able to personalize the Wellness Plan and found it relevant for their continued use of the app. Finally, users emphasized the importance of coaching support. They found the coach supportive and the calls convenient in terms of their schedule and appropriate length. Most users indicated that they were motivated by the coach to review intervention content and change their behaviors after coach calls.

**Table 6:** Pilot study: usability questionnaire (n=11).

Section and usability type		Question	DSD^a^	ASA^b^
**Overall**				
	Ease of learning	The terminology used in this application is clear.	0	91
	Ease of use	I was able to complete my tasks in a reasonable amount of time.	0	91
	Satisfaction	The content of the application met my expectations.	9	73
**Foundations**				
	Ease of learning	I found the lessons easy to understand.	0	100
	Ease of use	The lessons were about the right length.	0	82
	Usefulness	I found the lessons relevant to me.	0	73
**Wellness Plan**				
	Usefulness	I found the Wellness Plan relevant to me.	0	91
	Ease of learning	I found the Wellness Plan easy to understand.	0	82
	Usefulness	I liked being able to personalize the Wellness Plan.	0	82
	Usefulness	I learned something new from using the Wellness Plan.	0	73
	Usefulness	Having and using my personal Wellness Plan was useful for me.	0	73
**Daily Check-in**				
	Ease of learning	I found the Daily Check-in easy to use.	0	100
	Usefulness	Using the Daily Check-in made me more aware of how much I was sleeping.	0	91
	Usefulness	Using the Daily Check-in made me more aware of symptoms and early warning signs.	9	82
	Usefulness	I found using the Daily Check-in helpful.	0	73
**Daily Review**				
	Ease of learning	I found the Daily Review easy to understand.	0	100
**Coach**				
	Usefulness	I found the coach supportive.	0	91
	Usefulness	I found the coach calls useful.	0	91
	Ease of use	The coach calls were an appropriate length.	0	91
	Ease of use	I was able to schedule the coach calls at times that were convenient for me.	0	91
	Usefulness	I got more out of the application by working with the coach.	0	82
	Usefulness	I found the coach's role beneficial to my use of the application.	0	82
	Usefulness	Having the coach calls motivated me to read the lessons.	0	73
	Usefulness	I was motivated to make a change after phone calls with the coach.	0	73
**Psychiatrist**				
	Usefulness	Using LiveWell helped me communicate with my psychiatrist about how I was doing.	27	27
**Technical**				
	Usefulness	Once I completed my daily LiveWell activities, the reminders stopped appearing.	27	36
	Satisfaction	I found the reminders irritating.	45	27
	Usefulness	The reminders came on schedule as I programmed them to.	36	18
	Usefulness	I relied on the reminders to complete my daily LiveWell activities.	27	18
	Ease of use	The battery life of the phone was adequate.	55	9
	Ease of use	The study team was helpful and responsive to my technical issues.	0	82

^a^DSD: disagree/strongly disagree.

^b^ASA: agree/strongly agree.

Thematic analysis of exit interviews identified 6 usability themes: ease of use, ease of learning, usefulness, barriers, suggestions, and technical limitations (Multimedia Appendix 10). Users indicated that they found the app easy to use.

It was pretty clear what was going on. . . . The Daily Check-in was very easy. It took very little time. It [Daily Review] very quickly gave me information on what it thought was going well. (2005)

Some users reported that having self-management resources on their phones was especially convenient when they experienced symptoms. “It’s sort of like having a therapist in your phone” (2041).

Users found the Foundation lessons and Wellness Plan relevant and useful. They stated that they liked being able to personalize their wellness anchors and plans and that this process made their app use more meaningful.

We personalized [the Wellness Plan], going through each mood variation level and noting what I’ve personally experienced. At first, it was really generic, so going through it and having it be like “if you are up a level, this is what you are going to be seeing” . . . It’s my plan, not just a generic one. (2016)

Users also stated that personalizing their information inspired self-awareness.

Personalizing the information was really helpful, within the Wellness Plan, within triggers . . . It made me so much more aware of myself. (2066)

Moreover, users felt that the coaching support was motivating, useful, and helped them get more out of their app use.

I found the coaching really helpful. . . . [It] was helpful for motivation and for answering questions because the app was confusing at the beginning. (2061)

Users offered many suggestions about changes or additions to data viewing, navigation, personalization, and monitoring, such as adding tracking for energy level, alcohol use, diet, and exercise. “There should be more visual things . . . like video clips” (2063).

The one missing [from the Daily Check-in] is energy level. It’s very critical for bipolar. (2008)

Users noted the app’s navigation could be complicated, which sometimes led to not using parts of the app; however, coaching support helped resolve navigation difficulties. “Navigating was a little hard to get used to. . . . I’m not sure I ever looked at [the toolbox]” (2041).

It [Navigation] got easier. Like I didn’t get at first how you could put things in my toolbox, and I mean [my coach] explained it all to me. (2086)

Users still found that the Daily Review feedback could be repetitive if they were consistently experiencing problems with one specific behavioral target (eg, routine). In some cases, this repetitive feedback led to a discontinuation of use. “I got fed up after a couple weeks ‘cause [the Daily Review] was the same thing” (2063).

Some users felt that technical issues impeded their use of the app. Specifically, users experienced problems with the reminders to check in, which contributed to irritation and reduced reliance on the reminders.

Sometimes I can’t complete the [Daily Check-in] until later in the day, so pops up, I hit yes . . . and then it keeps popping up until it goes through all the reminder times that you missed. (2003)

Other users expressed difficulties with study equipment, such as frustration with poor phone battery life. “I had some difficulty with the phone. [It] would die all the time” (2016).

However, most users felt that the study team was helpful and responsive in resolving technical issues.

[The] technical things I brought up, [the coach] was like, “That’s been a problem we are working on it.” It’s good to know that it’s the phone and not me. (2005)

### Anchors

A thematic analysis of users’ mood, thoughts, and wellness rating scale anchors was completed to explore what types of signs and symptoms are relevant for monitoring wellness. The anchors for the mood, thought, and wellness rating scales were coded into subtypes and types (Multimedia Appendix 11). When the field trial anchors were formally coded, only 46% of the mood scale’s anchors were coded as “mood,” while the remainder were coded as “cognition,” “physical,” “behavior,” or “motivation.” For the thought scale, 69% of the anchors were coded as “cognition,” with the remainder coded mostly as “mood” and “behavior.” In contrast, when users identified personal anchors for the pilot study wellness scale (Table 7), the anchor types were coded as “behavior” (38%), followed by “cognition” (28%), “mood” (17%), “physical,” (10%) and “motivation” (7%). In terms of subtypes, anchors for thought content (17%), sleep (12%), thought process (10%), negative mood (10%), energy (9%), and social interactions (8%) accounted for two-thirds of the subtypes.

**Table 7:** Pilot study: thematic analysis of personalized wellness rating scale anchors (n=11).

Type (%) and subtype (%)			Personalized anchor (user ID)
**Behavior: visible activities or timing of such activities (38)**			
	Sleep: quality, duration, timing, need (12)	Stay up all night (2001) Oversleeping (2003) Nocturnal (2016)	
	Social: nonaggressive interactions with other people (8)	Enjoy seeing people (2005) More social (2016) Avoiding family & friends (2041)	
	Risky: increasing risk of injury or harm (4)	Buying things I don’t need (2003) Running traffic lights (2008) Driving too fast (2041)	
	Self-care: eating, drinking, grooming, hygiene, medications (4)	Well-fed (2001) Grooming, not shaving (2008) Forgetting meds (2016)	
	Leisure: for relaxation or enjoyment, including over engagement (3)	Making art (2003) Watching movies (2008) Exercising, walking (2041)	
	Speech: rate, rhythm, or volume of speech (3)	Less talkative (2016)	
	Work: employment, school, home care, volunteering (2)	Productive (2001) Study for 3-5 hours (2008) Going to work and school (2016)	
	Aggression: physical or psychological harm to person, object, or self (1)	Actively trying to harm self (2003) Super aggressive (2005) Self-harm (2016)	
	Substance: ingestion of psychoactive substances (1)	Drinking to dangerous excess (2001)	
**Cognition: acquiring knowledge and understanding through thought, experience, and senses (28)**			
	Content: what one is thinking about (17)	Life is not worth living (2001) Have lots of new ideas (2003) Odd thoughts (2008)	
	Process: logic, organization, coherence, and speed of thinking (10)	Faster thinking (2003) Bad judgment (2005) Problems with any decision (2041)	
	Perception: sensory processing, disturbances of sensory processing (1)	Hallucinations (2003)	
**Mood: emotional or affective state (17)**			
	Negative: unpleasant, disagreeable, lack of pleasure (10)	Irritable (2003) Persistent crabbiness (2005) Sad mood, “feeling down” (2041)	
	Positive: good, affirmative, or constructive (6)	Things are so exciting (2001) Laugh (2005) Easier to smile (2041)	
**Physical: relating to the body (10)**			
	Energy: strength and vitality (9)	Feel fatigued (2001) More restless (2008) Restless (2016)	
	Appetite: desire for food (1)	Not hungry (2001) Force self to eat (2005)	
**Motivation: reasons or drive to engage in behavior (7)**			
	N/A^a^	Willing to try anything (2001) Loss of interest (2008) Easier to maintain a routine (2041)	

^a^N/A: not applicable.

## Discussion

This paper describes the user-centered process that guided the development of the technological and human support components of LiveWell. Consistent with the importance of monitoring [3,11,25,60], the core of the LiveWell intervention consists of a daily self-monitoring tool, the Daily Check-in, which underwent multiple revisions during development. Changes to the technology and the coaching support were also made to help users set clear target goals, track the achievement of those goals, make adjustments, and communicate with care providers.

The initial Daily Check-in included both mood and thought rating scales. Coaches helped users develop personalized anchors for these scales to help users identify early warning signs and symptoms of an episode based on their past experiences. Examining these anchors revealed that the users’ anchors often did not fit within the bounds of the requested mood and thought categories. This suggested that users were being asked to personalize scales that did not capture their most relevant wellness variation experiences. Therefore, the mood and thought scales were collapsed into a single wellness rating scale. When users were provided with more freedom to identify personal anchors using the wellness scale, the anchors most commonly reflected behavioral—followed by cognitive, mood, physical, and motivational—signs and symptoms. More specifically, two-thirds of these anchors referenced thought content and process, sleep, negative mood, energy, and social interactions. These findings are consistent with the early warning sign literature indicating that individuals typically endorse changes in cognition (concentration, self-esteem, difficulty with decisions), behavior (more talkative and aggressive, changes in sleep duration), and energy before episodes [93,94].

Additional changes to the Daily Check-in were made to help users distinguish between getting adequate sleep and keeping a regular routine. Users reported that the Daily Check-in made them more aware of early warning signs and symptoms and how much they were sleeping. Early warning sign management and sleep duration are considered to be important targets that underlie the improved outcomes produced by empirically supported therapies for bipolar disorder [3,11,25,60]. It thus appears that user feedback led to changes in the Daily Check-in that may assist users in staying well.

Changes to the technology were also made to clarify goal setting, goal achievement tracking, and making adjustments (Daily Check-in and Daily Review), as users experienced difficulties with these strategies. In addition, changes were made to the Foundations and Toolbox to clarify the process of setting goals, making plans, monitoring, evaluating goal achievement, and making adjustments if needed. Due to the inconsistency with which users would act upon recommendations to contact their provider, pop-up notifications with a link to their psychiatrist’s phone number were added.

However, it was unclear that these technology changes were sufficient, so coaching roles were expanded to include support for self-management strategy use and clinical care communication. Research suggests that human support for app use reduces attrition and improves adherence; however, increased engagement does not always translate into improved outcomes [29,38,66,95-97]. Improved outcomes may arise from the inclusion of clinical support to ensure that users identify the content and tools relevant to their needs, use them correctly, and translate this use into their daily lives [38]. This suggests that expanding the coaching roles for LiveWell may improve outcomes. In creating the self-management and clinical care communication support roles for LiveWell, feedback from the coaches played an important role in developing these roles.

Users reported that working with their coaches to tailor their wellness rating scale anchors and plans made the intervention more relevant to their personal experiences. This feedback is consistent with prior studies indicating that personalizing application components to address user needs can increase user engagement and positively impact intervention outcomes [43,98,99]. Thus, the provision of generic plans was minimized to encourage the personalization of the wellness plans. Users noted that developing personally relevant plans motivated them to enact these plans. Taken together, these findings indicate that self-management interventions that utilize open-ended, personalized wellness scales and plans may help individuals develop insight into their health condition and more readily embrace and act on intervention content.

Finally, striking a balance between making the app easy to navigate and fulfilling participant requests for additional features was challenging. As user engagement typically decreases with challenging-to-use applications [100], an effort was made to prioritize user requests related to intervention functionality. Technical issues, such as problems with reminders and battery life, have been addressed with improvements in technology over time, such as the integration of app reminders into the Android operating system and smartphone battery-life improvements.

To support the ongoing improvement and dissemination of technology-based mental health interventions [38-42], we have provided a detailed description of the user-centered development process for LiveWell. This process suggests that individuals with bipolar disorder value target monitoring, personalization of goals and plans, and human support aids as self-management tools. In developing LiveWell’s technology and human support, feedback from both users and coaches played an important role, emphasizing the significance of engaging all stakeholders in intervention development. This attention to all-stakeholder input is broadly applicable to developing the technology and the human support for digital mental health interventions.

## Appendix

Multimedia Appendix 1 Field trial: scripts handouts.

Multimedia Appendix 2 Design sessions: coaching script handouts.

Multimedia Appendix 3 Design sessions: smartphone app mock-up.

Multimedia Appendix 4 Usability testing: script scenarios.

Multimedia Appendix 5 Usability testing: posttask questionnaire responses.

Multimedia Appendix 6 Pilot study: coaching scripts handouts.

Multimedia Appendix 7 Pilot study: user personalized plans.

Multimedia Appendix 8 Pilot study: exit interview script.

Multimedia Appendix 9 Pilot study: exit questionnaire responses.

Multimedia Appendix 10 Pilot study: exit interview thematic analysis.

Multimedia Appendix 11 User personalized anchors thematic analysis.

## Abbreviations

CBITS: Center for Behavioral Intervention Technologies
DSM-IV: Diagnostic and Statistical Manual of Mental Disorders–fourth version
F2F: face-to-face
