# Supplementary material for: A Smartphone-Based Self-management Intervention for Bipolar Disorder (LiveWell): User-Centered Development Approach
Source: JMIR Ment Health. 2021 Apr 12;8(4):e20424. doi: 10.2196/20424 (PMC8076988; doi:10.2196/20424)
Supplement: Multimedia Appendix 1 [file mental_v8i4e20424_app1.pdf]

LiveWell Field Trial: Application Training

Study ID:

Training Date:

Introduction

Hi, my name is ____________. I am going to be walking you through this phone training session today.

At this time, I just wanted to remind you that this training session is being video recorded. This is being done just to make sure that everyone is doing their job correctly.

Before we begin, let's take a moment to briefly go over what we will be doing today.

We will be reviewing the various features of the LiveWell phone tools that will be used in the study. As we go through these exercises together, you will be entering your responses directly on a test phone. If you are unsure of anything as we go through the training, please don't hesitate to ask questions.

Do you have any questions before we get started?

____

____

____

Great. Let's get started. (HAND PHONE TO PARTICIPANT)

General Instructions on Using Smartphone

Before reviewing the phone tools specific to our study, I want to take the first few minutes to go over some of the more basic features of the phone.

How much experience do you have using smartphones?

____

____

____

LiveWell Field Trial: Application Training

As I go through this basic training, feel free to let me know if I'm going too fast or not fast enough.

- **Power button:** The power button is on the top-right side of the phone. To shut down the phone, simply press and hold the Power button for a few seconds. Then choose Power off option from the menu. Now your phone has been turned off. To turn the phone back on, just press and hold the Power button again. Now this time only press the power button for a split second, the screen goes black and the phone goes into sleep mode (CLICK ON POWER BUTTON). To come out of sleep mode, just press the Power button again to turn the screen back on and return to the home screen.

  ____

  ____

  ____

- **Screen lock:** After turning the phone back on, you will notice that the screen is locked. At the bottom of the screen are instructions to 'Swipe Screen to Unlock.' You will be asked to unlock your screen every time your phone is turned on or when it's idle for a certain period of time. To unlock the screen, simply swipe or drag your fingers vertically in either direction across the screen.

  ____

  ____

  ____

- **Volume button:** The volume button is located on the top-left side of the phone: press the top of the button (marked '+') to increase the volume and the bottom of the button (marked '-') to lower it. You can use these buttons to adjust your volume at any time.

  ____

  ____

  ____

- **Home screen:** At the bottom center of your phone, you will see a rectangular shaped button. Press this button at any time to return to your home screen.

At the bottom of the screen there are also four icons (phone, internet, messaging, and apps).

Go ahead and click the ‘Phone’ icon. At the top of the screen you will now have several options, such as ‘Keypad’, ‘Logs’, and ‘Contacts’. To dial a number, click on ‘Keypad’ and a number pad should pop up. You also have the option to make phone calls or send messages through your contact book (CLICK ON CONTACTS). In addition, you can view all your incoming, outgoing, and missed calls though your phone ‘Log’.

Go ahead and return to the Home screen.

Above the Home button (at the bottom of the screen) you will also see the icon for ‘Internet’. Select this icon to open up your web browser (CLICK ON INTERNET).

Finally to the left of the ‘Internet’ icon, is the icon for ‘Messaging.’ Please go ahead and click this icon. This is where you will be able to send and receive text messages.

- **Back button:** To the right of the rectangular ‘Home’ button, is the “Back’ button. This button is not visible until you touch the surface of the phone. Go ahead and tap to the right of the ‘Home’ button and you should see a reverse arrow light up. This feature

allows you to return to your previous page regardless of what else you have going on at the time.

Do you have any questions before we move on?

Open LiveWell Application

On the bottom right hand side of the screen, you will see the icon for ‘Apps’. Go ahead and click that icon (CLICK ON APPS) to view the different applications installed on your phone.

Please go ahead and click the ‘LiveWell’ icon located on your screen to open the application (CLICK ON LIVEWELL).

You should now see the LiveWell home screen. This is the main menu of the application where you can view the various tools and tasks of the LiveWell system.

Open Instructions and Settings Menu

Inside the home page, please click on the button for ‘Instructions & Settings’. Under 'How to use this app', you will see three buttons: 'Sleep Times’, ‘Mood’, and ‘Thoughts.' You can click on these buttons to get information about how to complete the daily check-in for ‘Sleep Times’, ‘Moods’, and ‘Thoughts’.

Open and Review Instructions for Daily Sleep Check In

We will start by reviewing sleep times. Please click on the first button (CLICK ON SLEEP TIMES) that says 'Sleep Times'. During the course of the study, you will be asked to check in with the phone each morning. As part of the Daily Check In, you will be asked to indicate the time you went to bed last night with the intention of going to sleep, and the time you woke up that morning with the intention of starting your day. As this time, please take a moment to review the instructions for Daily Sleep Check In.

Open and Review Instructions for Daily Mood Rating

Now let's return to the survey instructions page using the back button to access a separate set of instructions for completing the daily mood rating. You can access the instructions (CLICK ON MOOD) by clicking on the button for Mood.

Before you begin charting your mood and thoughts, we are going to take some time to create a rating system that is personalized to your own symptoms and experiences.

Variations in mood and emotions are a normal part of life. However, with bipolar disorder the highs and lows are outside what is normal and cause problems. You will use a rating system that ranges from -3 for extremely depressed or apathetic all the way to +3 for extremely elevated or irritable.

In this instructions page, as well as at the bottom of the mood rating worksheet, you will find some more general descriptions of each rating to guide you in this process. Before we get started, please take a few moments to familiarize yourself with the rating system we will be

using. Also, as we complete the worksheets together, please feel free to refer back to this scale at any time.

____

____

____

<u>Open and Review Instructions for Daily Thought Rating</u>

Please return to the survey instructions page using the back button to access a separate set of instructions for completing the daily thought rating. You can access these instructions (CLICK ON THOUGHTS) by clicking on the button for Thoughts.

During the course of the study, you will be asked to complete a daily rating for speed and content of your thoughts.

Variations in thoughts are a normal part of life. When manic, excited, or under the influence of stimulants like caffeine the flow of thoughts is faster than usual. When depressed, discouraged, or under the influence of sedatives the flow of thoughts is slower than usual. You will use a rating system that ranges from -3 for extremely slow all the way to +3 for extremely fast.

Similarly, the content of your thoughts or ideas can range from -3 for seriously negative or +3 for seriously positive.

In the instructions page for Thoughts, as well as at the bottom of the page, you will find some more general descriptions of each rating to guide you in this process. Before we get started, please take a few moments to familiarize yourself with the rating system we will be using. Also, as we complete the worksheet together, please feel free to refer back to this scale at any time.

____

____

____

<u>Complete Mood and Thought Rate Worksheets</u>

- How do you feel when **well (0)**? Tell me what that's like for you.

  ____
  ____
  ____

  Now that we have done this, write down a few key words to describe your mood at these times. This will be a "0".

  ____
  ____
  ____

  Now let's talk a little about your thoughts. What is your thought speed at this time? This will be a "0".

  ____
  ____
  ____

  What about the content of your thoughts at this time? This will be a "0".

  ____
  ____
  ____

- Now think of a time when you felt your **lowest, or most depressed (-3)**. Tell me what that's like for you.

  ____
  ____
  ____

  Now that we have done this, write down a few key words to describe your mood at these times. This will be a "-3".

What about your thought speed at this time? This will be a “-3”.

What about the content of your thoughts at this time? This will be a “-3”.

- Now think of a time when you felt **slightly down (-1)**. Tell me what that’s like for you.

Now that we have done this, write down a few key words to describe your mood when slightly down. At these times your mood is understandable and manageable. Your mood is explainable by recent or upcoming events. This will be a “-1”.

What about your thought speed at this time? Your speed of thoughts is explainable by how you feel about recent or upcoming events, or by something you consumed. This will be a “-1”.

____

____

____

What about the content of your thoughts at this time? This will be a “-1”.

____

____

____

- Now tell me what it’s like for you at a **“-2”**.

____

____

____

Now that we have done this, write down a few key words to describe your mood at these times. This will be a “-2”.

____

____

____

What about your thought speed when slightly down? This will be a “-2”.

____

____

____

What about the content of your thoughts at this time? This will be a “-2”.

- Now think of a time when you felt your **highest, or most manic (+3)**. Tell me what it's like for you. This will be a "+3".

  Now that we have done this, write down a few key words to describe your mood at these times. This will be a "+3".

  What about your thought speed at this time? This will be a "+3".

  What about the content of your thoughts at this time? This will be a "+3".

- Now think of a time when you felt slightly up. This will be a "+1". Tell me what that's like for you.

Now that we have done this, write down a few key words to describe your mood when slightly up. At these times your mood is understandable and manageable. Your mood is explainable by recent or upcoming events. This will be a “+1”.

____
____
____

What about your thought speed at this time? Your speed of thoughts is explainable by how you feel about recent or upcoming events, or by something you consumed. This will be a “+1”.

____
____
____

What about the content of your thoughts at this time? This will be a “+1”.

____
____
____

- Now tell me what it’s like for you at a **“+2”**.

____
____
____

Now that we have done this, write down a few key words to describe your mood at these times. This will be a “+2”.

____

____

____

What about your thought speed when slightly down? This will be a “+2”.

____

____

____

What about the content of your thoughts at this time? This will be a “+2”.

____

____

____

<u>Open and Complete Daily Check-In</u>

Now that we have reviewed the various instruction pages, you are ready to complete a daily check in. At this time, please return to the application’s home page. Please click on the daily check-in (CLICK ON DAILY CHECK-IN). This is the information that you will be asked to fill in each morning.

____

____

____

Few reminders before you get started:

1) When charting your sleep, BEDTIME refers to the time you went to bed last night with the intention of going to sleep and WAKEUP refers to the time you got up this morning with the intention of starting your day.
2) When logging your daily mood thought ratings, please select the rating that best describes the way you feel <u>at this moment</u> using the worksheets we completed during this training session.

At this time, please go ahead and complete the daily check in and then click to save your responses and exit the LiveWell application (CLICK ON SAVE & EXIT LIVEWELL).

____

____

____

Review Initial and Reminder Prompts

At various times during the training session, I mentioned that the phone would prompt you to complete either the daily check in or weekly survey. You may be wondering exactly what I meant by "prompts". Over the next few minutes, I want to take the time to review the prompting system of the phone application.

Each morning, a message will pop up on your screen asking you if would like to check in with LiveWell at this time. You will have the option to either say 'Yes' (which will direct you to either the daily check in page or weekly survey page) or 'No' if you are unable to complete the check in at that time. If the latter is the case (and you are unable to complete the check in), responding 'No' will close the pop-up message on your phone's screen. However, you will always have the choice to complete the check in at some later point in the day by clicking on the LiveWell icon and opening the application manually. Also, if you are unable to complete the check in after the initial message of the day, you will receive three additional messages at scheduled intervals throughout the remainder of the day reminding you to check in. As soon as you complete the daily check in, you will not receive any additional prompts for the remainder of the day. The same general prompting "rules" will apply for the weekly surveys as well, however you will only be prompted to complete the weekly surveys once every two weeks.

____

____

____

Open and Select Daily Check In Time

Because everyone has a different schedule, the system was also designed to allow you to select the time at which you would prefer to check in each morning. The time you choose as your designated check in time will be the time in which you will receive the first message of the day prompting you to check in. We are going to go ahead and set that time together right now.

____

____

____

Please return to your applications home page, click on 'Instructions and Settings' to open the instructions page for the daily check in. At the bottom of the page, you will see the option to select a daily check in time (CLICK ON SELECT DAILY CHECK IN TIME). At this time, go ahead and choose the time each morning when you would prefer to complete the daily check in. If during the course of the study, you would prefer to be prompted to check-in at a different time, you can always return to this page to change your preference.

____

____

____

Open and Review Weekly Survey Instructions

Now let's return to the survey instructions page to access a separate set of instructions for the weekly surveys. You can access the instructions by clicking on the buttons with the surveys name. Please take a moment to briefly review the instructions for 'Survey #1' and 'Survey #2'.

____

____

____

Open and Complete a Weekly Survey

Now let's return to the LiveWell homepage. Every week after your start date, you will be prompted by the phone to complete two brief questionnaires. The first survey is about depressed mood, and the second survey is about elevated mood.

Click on the button that says ‘Weekly Survey’. You can enter your responses directly into the phone and press next. At the completion of the surveys, you will be able to submit your responses. Please take the time to complete these surveys at this time.

____

____

____

Questions and Thank You

Do you have any questions for me?

____

____

____

____

____

Thank you for coming in today.

LiveWell Field Trial: Application Training

Summary: ____

____

____

____

____

____

____

____

____

____

____

____

____

____

____

____

____

____

Study ID: Date:

Mood Rating Scale

-3	-2	-1	0	+1	+2	+3
Serious Down	Moderate Down	Slight Down	Well	Slight Up	Moderate Up	Serious Up
1.<br>2.<br>3.<br>4.<br>5.	1.<br>2.<br>3.<br>4.<br>5.	1.<br>2.<br>3.<br>4.<br>5.	1.<br>2.<br>3.<br>4.<br>5.	1.<br>2.<br>3.<br>4.<br>5.	1.<br>2.<br>3.<br>4.<br>5.	1.<br>2.<br>3.<br>4.<br>5.

In the boxes above, please print 3 to 5 words that best describe your mood for each rating point on the scale.

A rating of 0 means that you are feeling well.

A rating of +1 or -1 means that you are feeling slightly up or down. At these times your mood is understandable and manageable. Your mood is explainable by recent or upcoming events.

A rating of +2 or -2 means that your mood is a bit extreme and is impacting your thoughts and behaviors in moderately problematic ways.

A rating of +3 or -3 means that your mood is extreme and impacting your thoughts and behaviors in seriously problematic ways.

Study ID: Date:

Thought Rating Scale

-3	-2	-1	0	+1	+2	+3
Serious Down	Moderate Down	Slight Down	Well	Slight Up	Moderate Up	Serious Up
1.	1.	1.	1.	1.	1.	1.
2.	2.	2.	2.	2.	2.	2.
3.	3.	3.	3.	3.	3.	3.
4.	4.	4.	4.	4.	4.	4.
5.	5.	5.	5.	5.	5.	5.

In the boxes above, please print 3 to 5 words that best describe the speed and content of your thoughts for each rating point on the scale.

A rating of 0 means that your speed of thought it neither fast nor slow. Your ideas are neither especially negative nor positive.

A rating of +1 or -1 means that your speed of thought is slightly faster or slower. Your ideas are slightly negative or positive. It is explainable by how you feel about recent or upcoming events, or by something you consumed.

A rating of +2 or -2 means that your speed of thought is a bit extreme and your ideas are moderately negative or positive. Your thoughts are impacting your mood and behaviors in moderately problematic ways.

A rating of +3 or -3 means that your speed of thought is quite extreme and your ideas are seriously negative or positive. Your thoughts are impacting your mood and behaviors in seriously problematic ways.

LiveWell Field Trial: Post Application Training Interview

Study ID:

Interview Date:

Introduction

In this next part, we will be asking you to share with us your thoughts about the proposed LiveWell system, including the study enrollment procedures and the phone training session you just completed.

Although we love hearing positive comments, it is actually most helpful for us to hear about any problems.

Please don't hesitate to tell us about the things you don't like.

____

____

____

Phone Training

Overall, how helpful was the training in introducing you to the LiveWell system?

____

____

____

Do you feel the training adequately prepared you to use the phone tools and tasks?

____

____

____

Is there anything you wish I had covered during the training that we didn't go over?

____

____

____

Was the material presented in a manner that was easy to understand?

____

____

____

Did you find that the amount of time for the training session was appropriate?

____

____

____

What aspects of the training could be improved?

____

____

____

If you were leaving to use the phone today, would you want a set of written instructions covering what we did today?

____

____

____

Any additional comments regarding today's phone training session?

____

____

____

Phone Tools and Tasks

Daily Check In:

(SHOW PARTICPANTS A PAPER COPY OF THE CHECK -IN OR USE THE PHONE FOR REFERENCE)

With regards to the Daily Check In, please tell me what you thought about:

LiveWell Field Trial: Post Application Training Interview

1) The general layout: ____

____

____

2) The color: ____

____

____

3) The text (size, color, font): ____

____

____

Anything else?

____

____

____

<u>To Bed and Out of Bed Times:</u>

Where the times to use for going to bed for the night and getting up for the day clear?

____

____

____

Was it easy to do?

____

____

____

Will the time ranges work for you?

____

____

____

Would there be any problems completing this task?

___

___

___

Mood and Thought Ratings:

Overall, what did you think of the mood and thought anchoring worksheets?

___

___

___

What did you think of the wording in the mood rating scale?

___

___

___

Was it too easy or too tough to understand?

___

___

___

How about for the thought rating scale?

___

___

___

Were any aspects of the rating worksheets unclear for you?

___

___

___

Do you think referring back to this worksheet will be helpful in completing your daily mood and thought ratings?

Weekly Surveys:

With regards to the weekly surveys, what did you think about:

1) The general layout:

2) The text (size, color, font):

In completing the surveys, were there any words you didn't understand?

What did you think of the wording in the survey questions?

Was it too easy or too tough?

Did it read smoothly or did any of it seem awkward?

Enrollment Procedures

Telephone interview:

How did you like doing the initial interview over the phone?

Anything else?

Website:

What did you think about the study website?

Was it easy to find?

Did you think the website provided adequate information about the study?

Did you experience any difficulty navigating to the consent and eligibility screener?

How did you feel about completing the consent online?

Would you be comfortable providing an electronic consent for release of health information using a secure online server (instead of completing a written consent during the initial face-to-face visit with the study psychiatrist)?

Preferences

Do you currently use your cell phone for reminders like alarms, messages for yourself, etc.?

How often would you like to be prompted if you forget your daily check in?

Is there a particular timing for reminders that would be helpful (like a specific time of the day)?

____

____

____

Daily Habits

Mobile phone:

Can you tell me how you typically use your cell phone throughout the day? [For example, I carry my phone in my bag to work with me and then have it on my desk during the work day. I typically have it in my pocket when I'm at home. What about you?]

____

____

____

Do you have any specific habits about charging your phone?

____

____

____

Where do you keep your charger?

____

____

____

How many chargers do you have and of what type (wall, car, etc)?

____

____

____

Watch:

Do you usually wear a watch when you go out?

Do you usually wear a watch when you are at home?

[If not, where do you keep your watch when you are at home?]

(SHOW PATIENT STUDY WATCH)

Would you be willing to wear a watch like this all day every day?

Is there anything that would get in the way of you wearing it all day every day?

For us to get data from this watch, we will need you to return to us by mail in a prepaid envelope that would be mailed to you with a replacement watch. Do you think you would have any problems returning the watch by mail each month?

Because of this, we are looking at watches and other wrist worn devices to collect activity data so we and you can keep track of your daily activity and sleep patterns. Could you please give us your thoughts about the following options?

[SHOW META WATCH, CHRONOS, AND PICTURES OF THESE AND OTHER OPTIONS]

Up by Jawbone: ____

____

____

____

____

MotoActv: ____

____

____

____

____

FitBit Flex: ____

____

____

____

____

MetaWatch: ____

____

____

____

____

Pebble: ____

____

____

____

____

Current Mobile Phone Device & Plan

Do you have a mobile phone? ____

If yes:

What kind of phone? And who is your service provider?

____

____

____

Details of current phone plan?

____

____

____

Does your phone come with a SIM card? ____________________________________

To make things simpler, we want participants to be able to keep the same number when they enroll in the study. I will briefly explain the two different options that we are considering. Please let me know your thoughts on each and which you would prefer.

1) You could use your own mobile phone during the study, and we could try installing the study applications directly on your phone. In this case, we would 100% cover the cost of an unlimited talk, data, and messaging plan for the duration of the study.
2) We would give you a study phone to use during the duration of the study. Similarly to the first option, we would 100% cover the cost of an unlimited talk, data, and messaging plan for the duration of the study. However, there is a possibility depending on your current phone model that we would not be able to transfer your contacts.

Closing

That is all the questions I have for you today. I would like to thank you for taking the time to participant in this interview. Your feedback is invaluable to us.

LiveWell Field Trial: Exit Interview

Livewell ID: ________________________Date: _______________________Interviewer: _________________

Introduction

We would like you to share with us your thoughts about using the *LiveWell* system.

Although we love hearing the positive comments, the information that is most helpful is about the problems.

Please don't hesitate to tell us about things that didn't work so well.

Overview

1. Overall, how would you describe your experience using the LiveWell application?

   ____

   ____

   ____

2. Was there anything in particular you liked or found useful about using the application?

   ____

   ____

   ____

3. Was there anything you found challenging, difficult, or not useful about using the application?

   ____

   ____

   ____

4. If you could change anything about the LiveWell application, what would you change?

   ____

   ____

   ____

5. Anything overall about being in this research study you did or didn't like?

   ____

   ____

   ____

Livewell ID: ______________________Date: _____________________Interviewer: ________________

Daily Check In

1. What was your overall experience with the daily check ins?

____
____
____
____

2. Can you walk me through your typical use of the daily check ins?

____
____
____

3. Did you find completing the daily rating of your mood and thoughts to be helpful?
   [Why or why not?]

____
____
____

4. Did you find that the +3 to -3 rating scale was sufficient in capturing your daily mood?
   [Did you find the +3 to -3 scale to be adequate in range? If no, why not?]

____
____
____

   How about in capturing your thoughts?

____
____
____

Livewell ID: ______________________Date: ______________________Interviewer: ________________

Daily Check In

5. Was keeping a daily log of the time you got up and went to bed helpful?
   [Why or why not?]

   ___
   ___
   ___

6. Did you experience any difficulties completing the daily check in?

   ___
   ___
   ___

7. Was there anything you did to make completing the daily check in easier?

   ___
   ___
   ___

8. Anything else about the check ins?

   ___
   ___
   ___

Weekly Surveys

1. What was your overall experience completing the weekly surveys?

   ___
   ___
   ___
   ___

Livewell ID: ______________________ Date: ______________________ Interviewer: ________________

Weekly Surveys

2. Did you have any difficulties completing the weekly surveys?

___

___

___

3. Were there any questions in particular that you found confusing?

___

___

___

4. Did completing the surveys on Sundays work with your schedule?
   [If not, what would your preference have been?]

___

___

___

5. Anything else about the weekly surveys?

___

___

___

Reminders

1. What was your overall experience with the daily reminders?

___

___

___

___

Livewell ID: ________________________Date: _______________________Interviewer: _________________

Reminders

2. Were the daily reminders useful?

____
____
____
____

3. Was there anything about the daily reminders you didn't like?

____
____
____

4. Were the number of reminders ok? [Too many or not enough?]

____
____
____

5. How about the frequency of the reminders?
   [Too often or not often enough?]

____
____
____

6. Were the available options for preferred check-in time okay with your schedule?
   [If not, when would you have preferred to check in?]

____
____
____

Livewell ID: ______________________Date: _____________________Interviewer: ________________

Reminders

7. Did your preferred check-in time change at any point during the study (i.e. did you change this time under settings)? [If yes, what prompted you to do this?]

____

____

____

8. On a typical day, did you find yourself completing the check in after the first reminder message? [Or did you typically respond to prompts later in the day?]

____

____

____

9. Was there anything that would have made the reminders easier or less invasive for you?

____

____

____

10. Anything else about the reminders?

____

____

____

Use of Study Equipment

1. Can you walk me through your typical use of the study phone? [Where did you keep it, how frequently did you check it, etc.?]

____

____

____

Livewell ID: ________________________Date: _______________________Interviewer: _________________

Use of Study Equipment

2. Did you have any difficulties carrying the phone every day?

___

___

___

3. Did you have any difficulties wearing the watch all day every day?

___

___

___

4. Did you find having to change watches each month to be burdensome?
   [If yes, is there anything staff could have done to make this process easier for you?]

___

___

___

5. Was there anything you wish you'd had during the study to make it easier (extra phone chargers, car chargers, etc)?

___

___

___

6. Anything else about the study equipment?

___

___

___

___

Livewell ID: _______________________Date: ______________________Interviewer: ________________

Technical Issues

1. Did you experience any technical issues with the phone (i.e. trouble with reception, battery life, receiving and responding to prompts, completing daily check-ins and/or weekly surveys, etc.)?

   ____
   ____
   ____

   If yes, did you feel like staff was responsive in helping you address these issues?

   ____
   ____
   ____

2. Was the battery life ok?

   ____
   ____
   ____

Privacy

1. Did you have any concerns about your privacy while using the system? [If yes, describe].

   ____
   ____
   ____

Monthly Interviews

1. What was your overall experience with completing the monthly interviews (over the phone or in-person)?

   ____
   ____
   ____

Livewell ID: ______________________Date: ______________________Interviewer: ________________

Monthly Interviews

2. Did you experience any difficulties scheduling these interviews?

____

____

____

3. During these interviews, you were asked about symptoms of mania and depression for the past 2 weeks and for the 2 weeks prior.

   Did you experience any difficulty recalling symptoms for the past 2 weeks?

   ____

   ____

   ____

   How about for the 2 weeks prior?

   ____

   ____

   ____

4. Anything else about the interviews?

   ____

   ____

   ____

Thank you for your valuable feedback, is there anything else you'd like to add before we end?

____

____

____

____

____

____

Livewell ID: ________________________Date: _______________________Interviewer: _________________

Interviewer Comments

____

____

____

____

____

____

____

____
