# Supplementary material for: A Smartphone-Based Self-management Intervention for Bipolar Disorder (LiveWell): User-Centered Development Approach
Source: JMIR Ment Health. 2021 Apr 12;8(4):e20424. doi: 10.2196/20424 (PMC8076988; doi:10.2196/20424)
Supplement: Multimedia Appendix 2 [file mental_v8i4e20424_app2.pdf]

LiveWell: User Design Application Training

Study ID:_____________ Training Date: _____________ Trainer: _____________

Design Introduction

- Introduce self
- Here to take part in designing a phone application to help people manage BPD
- Application called LiveWell
- Still working on it and need to do testing to see if people like it

- As a volunteer, participation anonymous
- Take about 1 ½ hours
- If uncomfortable, you may end the interview at any time

Description of Design Evaluation

- Go over what we will do today
- Have you practice learning how to use the phone as if take home and use for next 4 months
- Application not ready yet – still building it
- Goal to get your input about what you like and don't like about the current ideas for *LiveWell*
- Feedback critical in helping us design an application that will be useful to you and others
- What you will see today are early designs
- Easy to change, make recommendation for changes you would like to see

- During session, next to you and recording your responses, questions, and feedback
- Use video to record responses for later review by the study team
- Only study team will view video

- Interview is to evaluate the *LiveWell* phone design
- I am not measuring your skills or abilities.
- Please remember: <u>*we are here to investigate the application, we are not testing you.*</u>

Design Instructions

- Interview consist of teaching you how to use *LiveWell*
- <u>think out loud</u> while we are doing this training
- Say out loud any thoughts or ideas that come to mind about application and training session

Do you have any questions right now?

____

____

Now start mock training [Make this clear]

LiveWell: User Design Application Training

Study ID:_____________ Training Date: _____________ Trainer: _____________

Introduction

Welcome. Thanks for coming to participate in the *LiveWell* study.

I'm _____________. I am a research assistant with training in psychology and have been working with the LiveWell team for over a year.

I will be working as your coach during your participation in the LiveWell program.

- Support your using the *LiveWell* phone application so most helpful for you
- Not provide therapy, but available to help with the application.
- You learn, practice skills for reducing and preventing symptoms by using application

- As talk today, take some notes, video record for review study team
- Only study team views video
- Use help us improve how teach to use application

- Today, about hour
- Go over how to use application
- May seem like a lot, don't worry about every detail
- More opportunities talk make sure getting most out *LiveWell* over 4 months.

Do you have any questions right now?

____

____

____

____

LiveWell: User Design Application Training

Study ID:_____________ Training Date: _____________ Trainer: _____________

Hopes

- Already provided us lot of information during enrollment.
- Today chance more of a conversation.
- Before going over *LiveWell* application
- Talk about what you hope to get out of program

[Use participant responses to emphasize how app will be useful to them. Build rapport. Support self-efficacy.]

Why did you decide to participate in this program?

____

____

____

____

What would you like to be different at the end of this program?

____

____

____

____

LiveWell: User Design Application Training

Study ID:_____________ Training Date: _____________ Trainer: _____________

LiveWell Program

Overview

- First little about ideas behind *LiveWell* phone application
- Then go over how to use application more detail
- Goal *LiveWell* program help decrease mood episodes

What do you do to avoid having mood episodes right now?

[Support participant's efforts to stay well.]

____

____

____

____

[Keep this overview brief.]

- *LiveWell* focuses medications and self-management as important ways of staying well
- Medications clearly shown reduce risk symptoms bipolar disorder.
- Most people know about the need for medications in bipolar disorder.
- Many do not know about self-management
- Self-management can cut down symptoms additional 50% compared with medications alone

- Program portable, easy to use
- Teach self-management strategies and help you take medications regularly
- Self-help program
- Help develop knowledge and skills
- Take work on your part, but get easier over time

Have you had any experience with self-management strategies for bipolar disorder?

[Make links to how LiveWell may be useful if possible.]

____

____

____

____

LiveWell: User Design Application Training

Study ID:_____________ Training Date: _____________ Trainer: _____________

Home Page

- Start by going over application, see how help you
- Let know going too fast/slow
- Ask questions any time

[Give participant the phone. Show where to go on the phone for application icon.]

[*For design, explain mock up for general sense of how would work. Back button. Use pink buttons to navigate.*]

Let's look at the home page for the *LiveWell* application.

You will see this home page when you open the application.

There are five buttons on the home page:

- Foundations
- Check Ins
- Daily Review
- Wellness Plan
- Instructions & Settings

Push a button to see content.

Go ahead and explore the application for a bit.

Any thoughts or questions before we look at the different parts of the application?

____

____

____

____

LiveWell: User Design Application Training

Study ID:_____________ Training Date: _____________ Trainer: _____________

Foundations

[Have participant navigate to the Foundations and go over the modules listed with them.]

- Contains basic information help learn about managing bipolar disorder.
- 8 modules

Push button for module to start reading.

- Each module take you about 5-10 minutes to read
- Read all the modules during the 1st month of the program
- About two modules a week 1st month

What do you think about this schedule?

____

____

____

____

[Elicit commitment. Find out about potential obstacles.]

- To help get the most out of modules:
- Brief 5-10 min phone call each week 1st month go over modules you read
- In 4th week, longer call about 20-30 minutes wrap up last modules

Any questions about these coaching calls?

____

____

____

____

[Worked toward mutual expectations for use of application and completion of lessons.]

LiveWell: User Design Application Training

Study ID:_____________ Training Date: _____________ Trainer: _____________

Check Ins

[Have user navigate to the Daily Check Ins page from the Home Page.]

- Go over daily, weekly check ins
- More detail later
- Talk briefly about them first

Check Ins: Daily

- Help keep track medication use, sleep, routines, mood and symptoms
- Take meds, regular sleep and routines, monitor for symptoms important self-management
- Also record how doing to help psychiatrist provide best treatment

Any thoughts or questions about the daily check ins?

____

____

____

____

[Have user navigate to the Weekly Check Ins page from the Home Page.]

Check Ins: Weekly

- Each week, complete 2 questionnaires measure symptoms depression, mania
- Good way to keep track how doing over time
- Help providers as standard measures depression, mania

These questionnaires will come up once a week on Sundays after you complete the daily check.

Any thoughts or questions about the weekly check ins?

____

____

____

____

LiveWell: User Design Application Training

Study ID:_____________ Training Date: _____________ Trainer: _____________

Check Ins: Prompts

- Recommend complete check ins same time each morning
- Sometimes hard to remember to check in
- Every day application displays a reminder to check in

- Reminder comes up on phone screen
- Ask if like to check in
  - Good time, say yes and complete
  - Not good time, hit no
  - Application remind you to check in again later

Check Ins: Settings

[Have patient navigate to the home button and show how can choose check in time]

- Set when like to receive first reminder day in settings and instructions

Any questions about the basics of check-ins or reminders?

____

____

____

____

- Now go over each part of daily check in more detail
- Start with medications

Check Ins: Daily – Medications

[Have participant navigate to the Daily Check In page]

- Taking medications helps decrease symptoms
- Can be difficult for people to take medications daily

Right now how do keep track of whether you took your medications?

[Support efforts. Elicit and highlight how application may be useful.]

____

____

____

[Go over how track medication use in application]

- Under medications in daily check in, 3 buttons: All, Some and None
- Record took all, some, or none daily psychiatric medications day before by push button

[Explain prn medications. Would still push all if don't use prn medications only routine meds count]

Do you think using this might help you keep track of and take your medications daily?

____

____

____

____

[Explore if think check in track took meds useful]

[Check if think they can remember took meds day before or not]

[Have list of current medications and briefly review correct. Ask call in if meds change.]

LiveWell: User Design Application Training

Study ID:_____________ Training Date: _____________ Trainer: _____________

Check Ins: Daily – Sleep

- Getting sleep important reduce symptoms, episodes
- Having regular bedtime/wakeup time important reduce symptoms, episodes
- So keeping track of your sleep very useful.

[Go over how track medication use in application]

- Push button to record when to bed the night before with the intention of going to sleep
- Select time, does not have to be when you went to sleep but when you tried to get to sleep

- Push button to record when you got up planning to start your day
- Select time you got up

Do you think this might help you keep track of and maintain regular sleep patterns?

____

____

____

____

[Explore if think check in track sleep useful]

Summary of Pittsburgh Sleep Quality Questionnaire

____

____

____

____

____

____

LiveWell: User Design Application Training

Study ID:_____________ Training Date: _____________ Trainer: _____________

<u>Check Ins: Daily – Psychoeducation: Mania, Depression, and Early Warning Signs</u>

- Before go over early warning signs and wellness rating portion daily check in:
- Go over symptoms of mania and depression
- Go over what early warning signs are
- Help us understand what we are talking about

<u>Check Ins: Daily – Psychoeducation: Mania</u>

Let's talk a bit about what manic episodes have been like for you.

Can you tell me some of your symptoms of mania?

___

___

___

Look at this hand out that covers the main symptoms of mania.

[Give out and look over handout 1: Symptoms of Mania.]

What do you think about this?

___

___

___

Summary of Affective Disorders Evaluation (Manic episodes)

___

___

___

___

___

LiveWell: User Design Application Training

Study ID:_____________ Training Date: _____________ Trainer: _____________

<u>Check Ins: Daily – Psychoeducation: Early Warning Signs of Mania</u>

- In addition mania, many people notice low-level symptoms/other signs before episode
- These kinds of changes are early warning signs
- Learn to notice take action avoid episodes

Can you tell me about any early warning signs that you have noticed before getting <u>manic</u>?

____

____

____

____

Let’s review the list of early warning signs of mania that are pretty common.

[Give out and look over handout 2: Early warning signs of mania]

What do you think about these?

____

____

____

____

I will load 3-5 early warning signs of mania onto the phone for you.

- Either check 3-5 early warning signs from list on the list or write down personal signs

[Have participant complete the early warning signs of mania handout.]

LiveWell: User Design Application Training

Study ID:_____________ Training Date: _____________ Trainer: _____________

Check Ins: Daily – Psychoeducation: Depression

Ok. Now let's talk about what episodes of depression have been like for you.

Can you tell me some of your symptoms of depression?

____

____

____

____

Ok. Now let's take a look at this hand out that covers the main symptoms of depression.

[Give out and look over handout 3: Symptoms of Depression.]

What do you think about this?

____

____

____

____

Summary of Affective Disorders Evaluation (Depressive episodes)

____

____

____

____

____

LiveWell: User Design Application Training

Study ID:_____________ Training Date: _____________ Trainer: _____________

<u>Check Ins: Daily – Psychoeducation: Early Warning Signs of Depression</u>

- Many notice low level symptoms or other signs well before entering a depressive episode
- These kinds of changes are early warning signs
- Learn to notice take action avoid episodes

Can you tell me about any early warning signs that you have noticed before getting <u>depressed</u>?

____

____

____

____

Here is a list of early warning signs of depression that are pretty common.

[Give out and look over handout 4: Early warning signs of depression]

What do you think about these?

____

____

____

____

I will load 3 or 5 early warning signs of depression onto the phone for you.

- Either check 3-5 from list or write down ones that make sense for you.

[Have participant complete the early warning signs of depression handout.]

LiveWell: User Design Application Training

Study ID:_____________ Training Date: _____________ Trainer: _____________

Check Ins: Daily – Early warning signs

- Each day check in, think about whether or not noticed any your early warning signs in last day

Under Early Warning Signs, press Down button see list your early warning signs of depression

- If present, push radial button next to it
- If absent, just leave them blank

Under Early Warning Signs, press Up button see list your early warning signs of mania

- If present, push radial button next to it
- If absent, just leave them blank

Don't have push Up and Down buttons and look at lists when not noticing any early warning signs

But, sometimes even if not noticing any early warning signs, good idea to review the lists

Do you have any questions about checking for early warning signs each day?

____

____

____

____

LiveWell: User Design Application Training

Study ID:_____________ Training Date: _____________ Trainer: _____________

Check Ins: Daily – Wellness scale

- Before chart your wellness, determine rating system that is right for you
- Create a system personalized to own experiences and symptoms

- Use what told me about your symptoms of mania and depression as well handouts to create personal wellness scale
- Use EWS check list to help

Now let's work on creating your own personal wellness scale. We will use a scale that goes from -4 for severely depressed to +4 for severely manic. Here are some handouts to help us with this.

[Give out and look over handout 5-6: Wellness Rating Scale Instructions and Fillable]

Let's look at the rating scale.

[Go over the 9 ratings]

Any thoughts about the wellness scale?

____

____

____

____

Check Ins: Daily – Wellness scale

- Go through and review each of 9 wellness ratings
- Ask you to write down 3-5 brief statements for each rating on the scale.
- Words can describe your moods and emotions, the kind of thoughts you have, whether your thinking is fast or slow, your behaviors, sleep, or energy levels for each rating

Have some reminders each rating meaningful to you well, depressed, or manic.

Will load anchoring words on phone, so keep each line short.

[Use information already provided to adjust prompting and cues for each rating]

Study ID:______________ Training Date: ______________ Trainer: ______________

Check Ins: Daily – Wellness scale

Let's start with when you are doing **well** and feeling **balanced (0)**.

Tell me what that's like for you.

____

____

____

____

Now write down a few words that describe you when you are doing well. **This will be a 0**.

____

____

____

____

<u>Check Ins: Daily – Wellness scale</u>

Now, a time when you felt your **lowest or most depressed (-4)**.

[If never suicidal, hospitalized or very severe depression, consider what lead them call 911 or go hospital]

May have:

- Been unable maintain your regular activities and routines
- Been unable to sleep, eat, or bath regularly
- Engaged in behaviors with serious consequences (risky, dangerous)
- Thought about suicide including making or acting on plans to kill yourself
- Experienced psychotic symptoms such as delusions or hallucinations
- Needed to be hospitalized.

Tell me what a very severe depression is like for you.

____

____

____

____

Now write down a few words that describe you at these times. **This will be a -4**.

____

____

____

____

Summary of Affective Disorders Evaluation (Depressive episodes)

____

____

____

<u>Check Ins: Daily – Wellness scale</u>

Study ID:_____________ Training Date: _____________ Trainer: _____________

Time when you felt **slightly down (-1)**. This type of down is

- Typical response to routine negative event in daily life
- Usually normal variation in mood.

Tell me what that's like for you.

____

____

____

____

Now write down a few words that describe you at these times. **This will be a -1**.

____

____

____

____

LiveWell: User Design Application Training

Study ID:_____________ Training Date: _____________ Trainer: _____________

Check Ins: Daily – Wellness scale

Time when you felt **mildly down (-2)**. With this type of down:

- Early warning signs may be present
- Some symptoms may be present
- Full blown episode may be coming
- Still maintain your usual activities and routines.

Tell me what that's like for you.

____

____

____

____

Now write down a few words that describe you at these times. **This will be a -2**.

____

____

____

____

LiveWell: User Design Application Training

Study ID:_____________ Training Date: _____________ Trainer: _____________

Check Ins: Daily – Wellness scale

Time when you felt **moderately down (-3)**. With this type of down:

- Multiple symptoms
- Symptoms likely continue day to day
- Full blown episode probably happening
- Difficult to maintain your usual activities and routines.

Tell me what that's like for you.

____

____

____

____

Now write down a few words that describe you at these times. **This will be a -3**.

____

____

____

____

Summary of Affective Disorders Evaluation (Depressive episodes)

____

____

____

Study ID:_____________ Training Date: _____________ Trainer: _____________

Check Ins: Daily – Wellness scale

Time when you felt your **highest or most manic (+4)**.

[If never hospitalized or very severe mania, consider what would lead them to call 911 or go to the hospital]

May have:

- Been unable engage in your daily activities and routines
- Been unable to sleep, eat or bath regularly
- Engaged in behaviors with serious consequences (risky, dangerous, fighting, promiscuous)
- Brought very expensive things you didn't need
- Had very poor judgment, made very bad decisions
- Experienced psychotic symptoms such as delusions or hallucinations
- Needed to be hospitalized.

Tell me what a very severe mania is like for you.

____

____

____

____

Now write down a few words that describe you at these times. **This will be a +4**.

____

____

____

____

Summary of Affective Disorders Evaluation (Manic episodes)

____

____

____

Study ID:_____________ Training Date: _____________ Trainer: _____________

Check Ins: Daily – Wellness scale

Now think of a time when you felt **slightly up (+1)**. This type of up is

- Typical response to routine positive event in daily life
- Usually normal variation in mood.

Tell me what that's like for you.

____

____

____

____

Now write down a few words that describe you at these times. **This will be a +1**.

____

____

____

____

LiveWell: User Design Application Training

Study ID:_____________ Training Date: _____________ Trainer: _____________

Check Ins: Daily – Wellness scale

Time when you felt **mildly up (+2)**. With this type of up:

- Early warning signs may be present
- Some symptoms may be present
- Full blown episode may be coming
- Still maintain your usual activities and routines.

Tell me what that's like for you.

____

____

____

____

Now write down a few words that describe you at these times. **This will be a +2**.

____

____

____

____

LiveWell: User Design Application Training

Study ID:______________ Training Date: ______________ Trainer: ______________

Check Ins: Daily – Wellness scale

Time when you were **moderately up (+3)**. With this type of up:

- Have multiple symptoms
- Symptoms likely continue day to day
- Full blown episode probably happening
- Difficult to maintain your usual activities and routines.

Tell me what that's like for you.

____

____

____

____

Now write down a few words that describe you at these times. **This will be a +3**.

____

____

____

____

Summary of Affective Disorders Evaluation (Manic episodes)

____

____

____

LiveWell: User Design Application Training

Study ID:_____________ Training Date: _____________ Trainer: _____________

Check Ins: Daily – Wellness scale

How to complete wellness rating:

- General descriptions of wellness rating scale, personal anchoring words available review
- Show you where in a little while

- Each day record wellness rating by pressing the rating on daily check in
- Once press the number, turns white, know it has been selected

Do you have any questions about the daily wellness ratings?

____

____

____

____

Before we move on, do you have any questions about check ins?

____

____

____

____

LiveWell: User Design Application Training

Study ID:_____________ Training Date: _____________ Trainer: _____________

Daily Review

[Help participant navigate to the daily review page on the phone.]

- Similar to check ins
- Recommend look at every day
- Taken to after completing daily check in
- Get feedback after completing check in
- But can go to whenever you want

The daily review has 5 buttons:

- Medications
- Sleep
- Routines
- Wellness
- EWS for early warning signs

How it works:

- Quick daily summary of how you are doing in each of these areas
- If appears all is well, button green
- If some cause for concern, the button yellow

- If push yellow, questions about how doing, suggestions things you can do

- If push green, receive brief feedback
- Can push Learn More button

How might you find the daily review useful?

____

____

____

____

LiveWell: User Design Application Training

Study ID:_____________ Training Date: _____________ Trainer: _____________

Charts

[Have participant navigate to Daily Review and show s/he where the Charts button is.]

- Button at bottom of daily review
- More detailed look at patterns that occur over time

- Summary of medication, sleep, routine, EWS, and wellness for a week or a month
- Green for day if going well
- Yellow for day if some problems

- Also be able to access more detailed information
- For example, how sleep and wellness rating are related

Any thoughts about how the charts might be useful to you?

____

____

____

____

LiveWell: User Design Application Training

Study ID:_____________ Training Date: _____________ Trainer: _____________

Provider Info

Your psychiatrist will be able to review a report that gives a weekly overview of how you are doing.

[Give out handout 7 Provider Report. Review with participant.]

[Elict any information would or would not want sent to provider. Address secure.]

Psychiatrist may also receive an email alert.

Any time psychiatrist receives email alert, application recommend that you call your psychiatrist.

Do you have any questions about the information that *LiveWell* will send to your provider?

____

____

____

____

Wellness Plan

[Have participant navigate to the Wellness Plan.]

You will learn about developing a wellness plan in module 8 of the Foundations section.

You and I will talk on the phone and personalize the wellness plan for you.

Right now let's look at a standard plan for effectively managing bipolar disorder.

The wellness plan has three sections

- My Resources
- Reduce Risk
- Awareness/Action

To view each section of the wellness plan, you just press the tab on the phone screen.

Go ahead and explore this standard wellness plan.

LiveWell: User Design Application Training

Study ID:_____________ Training Date: _____________ Trainer: _____________

Wellness plan – My Resources

[Have participant navigate to My Resources]

In My Resources, buttons for:

- Medications
- Skills
- Team

[Have participant press the Medications button]

Medications:

- Press button, take to list of medication currently taking for bipolar disorder
- Always update list by calling me with changes to medications
- I load changes into application

[Have participant press the Skills button]

Skills:

- Press skills button, list of skills for managing stressors and symptoms
- Learn more about skills by reading the Foundations modules
- Can look over list anytime, use when seem appropriate

[Have participant press the Team button]

Team:

- Addition to medications and using skills, effective management requires good team in place
- Press Team, list of people who can support you in doing well
- Includes psychiatrist and people like therapist, friends or family members who support you

Do you have any thoughts or questions about the resources section of the wellness plan?

____

____

____

____

LiveWell: User Design Application Training

Study ID:_____________ Training Date: _____________ Trainer: _____________

Wellness plan – Reduce Risk

[Have participant navigate to Reduce Risk]

Triggers can increase your risk of experiencing symptoms.

Reduce risk of triggers and symptoms by maintaining a healthy lifestyle.

You will learn more about this in the Foundations module 4.

[Give participant handout 8: Reduce Risk Recommendations. Review]

You can see right now some common recommendations for maintaining a healthy lifestyle.

During week 4, talk on phone and I will load your personal plan for maintaining a healthy lifestyle.

Any thoughts or questions about the reducing risks section of the wellness plan?

____

____

____

____

[Review sections of the BASICs as needed]

The BASICs for maintaining a healthy lifestyle are broken down into 5 areas here:

- **Blueprint** your day to maintain regular routines of activities and sleep
- **Abstain** or moderate your use of alcohol and other substances
- **Sleep** enough
- **Interact** with others in enriching ways
- **Calm** is the aim, manage stressors

<u>Wellness plan – Being aware and taking action</u>

The last section of the wellness plan has two parts.

First Part:

- **Being aware**
- Look-out for stressors, early warning signs, and keeping track of how you are doing

Second Part:

- **Taking action**
- Plan for what do when you well or when having slight, moderate, or severe symptoms

[Review standard recommendations. Handout 9]

- Right now wellness plan standard suggestions to do for each situation
- Later you and I will update this with your own personal plan

Do you have any thoughts or questions about awareness/action section of the wellness plan?

____

____

____

____

LiveWell: User Design Application Training

Study ID:_____________ Training Date: _____________ Trainer: _____________

Instructions

[Show subject that when push instructions button in Settings & Instructions these buttons available]

- Covered a lot today
- Instructions section also goes over what talked about today for each section

[Have navigate to schedule]

- Will have a schedule, give list of times recommend complete different modules
- For any planned phone calls, I will load dates and times into schedule

- Place where you can find out about appointments and timing of program
- Recommend you also keep dates in regular calendar or schedule as well

Do you have any questions about the settings and instructions section of the application?

____

____

____

____

Study ID:_____________ Training Date: _____________ Trainer: _____________

Summarize

What it's designed to do:

- Help you learn better manage bipolar disorder
- Less likely have symptoms, better manage symptoms when they occur

Important clarifications:

- Not a replacement for working with your psychiatrist and other mental health providers
- Not an emergency service
- Urgent psychiatric problem, contact your psychiatrist immediately
- Although provides summary information, not a replacement for talking with your psychiatrist about severe symptoms or even moderate symptoms that don't go away in a day or two

Also, if you are feeling suicidal, you should call 911 or go to the nearest emergency room.

LiveWell: User Design Application Training

Study ID:_____________ Training Date: _____________ Trainer: _____________

Coaches Role

- Help learn how to use the application so most helpful for you
- Set up phone calls to see how things are going
- Opportunity for you to ask questions about application
- Can also contact me by email

- Review any difficulties you've been having with application
- Scheduled calls once a week in 1st month while complete Foundations modules
- Calls about 5-10 minutes
- Week 4 longer calls to wrap up modules and develop personalized wellness plan

Does this sound OK?

____

____

____

____

Any questions?

____

____

____

____

LiveWell: User Design Application Training

Study ID:_____________ Training Date: _____________ Trainer: _____________

<u>Program Participation Barriers and Problem Solving</u>

Beyond concerns talked about [name them], **what might make hard participate *LiveWell*?**

____

____

____

____

<u>Commitment and Goals</u>

[Summarize participant's story, dilemmas/strengths, barriers/solutions]

[Highlight "change talk" and perceived positives of LiveWell program. Link to hopes.]

Request:

- Read 2 modules a week during first month
- Use daily check in and daily review every day for next 3 months
- Review Wellness Plan routinely and practice lifestyle and coping skills

As coach, I track your use of the application and support you in getting the most out of it.

When do you think you will start using the *LiveWell* application?

____

____

____

____

How often do you think you can use the *LiveWell* application each week?

____

____

____

____

Study ID:______________ Training Date: ______________ Trainer: ______________

Finish Up

Ok, so we will talk next week.

I appreciate your taking such an active part in learning about the program today.

I really think you will find this program useful in helping you stay well.

Remember sometimes things come up and people aren't able to use the program as planned.

We can always talk about any problems and I'm happy to help.

Questions and Thank You

Do you have any questions for me?

____

____

____

____

Thank you for coming in today.

Summary: ____

____

____

____

____

____

____

____

____

____

____

Wellness Rating Scale

Wellness	Definition	Anchors
**+4 Severe Up**	Poor judgement. Dangerous behaviors Not sleeping. Hallucinations/delusions.	
**+3 Moderate Up**	Many symptoms day to day. Manic episode probably happening. Difficult to maintain activities/routine.	
**+2 Mild Up**	Some symptoms, early warning signs. Manic episode may be coming. Can still maintain activities/routine.	
**+1 Slight Up**	Response to recent/upcoming good event. Likely normal variation in wellness. Understandable and manageable.	
**0 Balanced**	Neither up nor down. Doing well.	
**-1 Slight Down**	Response to recent/upcoming bad event. Likely normal variation in wellness. Understandable and manageable.	
**-2 Mild Down**	Some symptoms, early warning signs. Depressive episode may be coming. Can still maintain activities/routine.	
**-3 Moderate Down**	Many symptoms day to day. Depressive episode probably happening. Difficult to maintain activities/routine.	
**-4 Severe Down**	Serious ideas about suicide. Immobilized. Dangerous behaviors. Disrupted sleep. Hallucinations/delusions.	

Symptoms of Depression

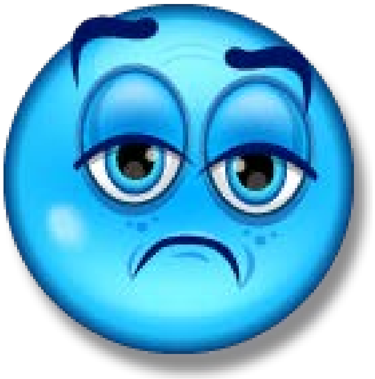

The hallmark of depression is **low mood or diminished interest and pleasure in life**. Other symptoms include:

- ✓ Weight loss or weight gain
- ✓ Sleeping too much or too little
- ✓ Physical agitation or slowing down
- ✓ Fatigue or loss of energy
- ✓ Feeling worthless or guilty
- ✓ Difficulty concentrating
- ✓ Thoughts of death or suicide

Low mood or diminished interest/pleasure plus 4 or more other symptoms on **most days for 2 weeks** is considered a full-blown depressive <u>episode</u>.

Early Warning Signs of Depression

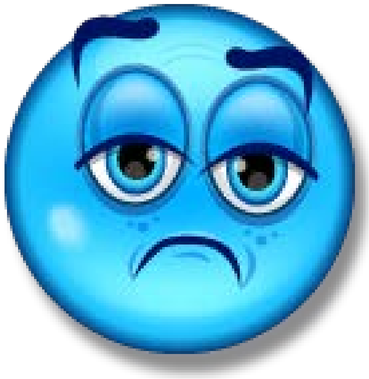

Many people notice low level symptoms or other signs well before entering a depressive episode. These are called early warning signs. Common early warning signs include:

- ✓ Sad or anxious mood
- ✓ Less energy than usual
- ✓ Problems concentrating
- ✓ Less interest than usual
- ✓ Negative thinking
- ✓ Withdrawn
- ✓ Sleep disturbance
- ✓ Guilt

How can you tell if an episode of depression is coming? What changes do you recognize in yourself that lead up to a depressive episode? Are there any signs that you notice **first**?

Symptoms of Mania

The hallmark of mania is a **euphoric or irritable mood**. Other symptoms include:

- ✓ Inflated self-esteem/grandiosity
- ✓ Decreased need for sleep
- ✓ More talkative than usual
- ✓ Racing thoughts
- ✓ Difficulties concentrating
- ✓ Increased activity level
- ✓ Risky activities

Elevated or irritable mood plus 3 or more other symptoms **for a week** is considered a full-blown manic episode.

Early Warning Signs of Mania

Many people notice low level symptoms or other signs well before entering a manic episode. These are called early warning signs. Common early warning signs include:

- ✓ Sleep disturbance
- ✓ More active than usual
- ✓ More talkative than usual
- ✓ More social than usual
- ✓ More irritable/agitated than usual
- ✓ Increased energy
- ✓ Increased self-esteem
- ✓ Racing thoughts

How can you tell if an episode of mania is coming? What changes do you recognize in yourself that lead up to a manic episode? Are there any signs that you notice **<u>first</u>**?
