# Supplementary material for: A Smartphone-Based Self-management Intervention for Bipolar Disorder (LiveWell): User-Centered Development Approach
Source: JMIR Ment Health. 2021 Apr 12;8(4):e20424. doi: 10.2196/20424 (PMC8076988; doi:10.2196/20424)
Supplement: Multimedia Appendix 3 [file mental_v8i4e20424_app3.pdf]

LIVEWELL

Foundations

Check Ins

Daily Review

Wellness Plan

Instructions & Settings

FOUNDATIONS

1 - Overview

2 - Basic Facts About Bipolar

3 - Using Medications

4 - Lifestyle Skills

5 - Coping Skills

6 - Using a Team

7 - Being Aware

8 - Taking Action

CHECK INS

Daily Check In

Weekly Check In

DAILY CHECK INS

Medications

All	Some	None

Went to bed

11pm

Got up

7am

EWS

Down	Up

Wellness

-4	-3	-2	-1	0	+1	+2	+3	+4

Save

Weekly Survey
Depressed Mood

Over the past week, how often have you been bothered by the following problems?

Little interest or pleasure in doing things

Not at all

Several days

More than half the days

Nearly every day

Next

QUESTION NUMBER IS: 1 OF 8

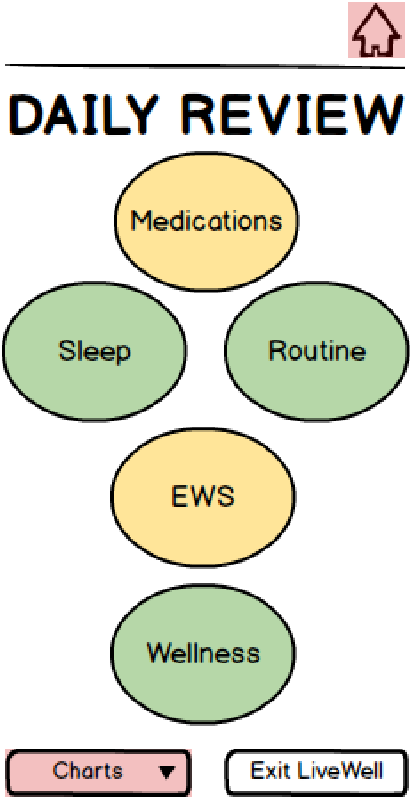
DAILY REVIEW
Medications
Sleep
Routine
EWS
Wellness
Charts
Exit LiveWell

My Medications

Name: Lithium
Amount: 600mg
When: Every morning

Name: Lithium
Amount: 300mg
When: Every night

Name: Lithium
Amount: 15mg
When: Every morning

Name: Lithium
Amount: 1.0 oz
When: At night, as needed for insomnia

CHARTS

Show Weeks

Show Months

AUGUST	1 Mon	2 Tue	3 Wed	4 Thu	5 Fri	6 Sat	7 Sun
Early Warning Signs							
Wellness							
Medications							
Sleep							
Routine							

Details

Widget with randomly rotating details graphs thumbnail here

WELLNESS PLAN

My Resources | Reduce Risk | Awareness & Action

My Medications

My Skills

My Team

WELLNESS PLAN

My Resources | Reduce Risk | Awareness & Action

Blueprint	Exercise at least three times per week<br>Continue attending weekly group therapy<br>Spend more time out of my house
Abstinence	Limit coffee intake to one cup per day<br>Avoid triggers that lead to smoking again<br>Limit drinking to special occasions
Sleep	Turn off TV in my bedroom<br>Force myself out of bed when I'm feeling down<br>Get at least 6-8 hours of sleep per night
Interpersonal	Attend social gatherings in my neighborhood<br>Spend more time with family<br>Limit interactions with negative coworkers
Calm	Get rid of clutter in my home<br>Create a payment plan for unpaid medical bills<br>Read more

WELLNESS PLAN

My Resources | Reduce Risk | Awareness & Action

Be Aware	Take Action
+4 Severe Up	Call your psychiatrist.<br>Call 911.<br>Go to the hospital.
+3 Moderate Up	Take medications daily. Use lifestyle and coping skills.<br>Use supports.<br>Call your psychiatrist.<br>Consider more intensive treatment.
+2 Mild Up	Take medications daily.<br>Use lifestyle skills.<br>Use coping skills.<br>Use supports. Work more closely with your psychiatrist.
+1 Slight Up	Take medications daily.<br>Watch closely.<br>Check for early warning signs.<br>Manage triggers with lifestyle skills.
+0 Balanced	Take medications daily.<br>Maintain a healthy lifestyle.
-1 Slight Down	Take medications daily.<br>Watch closely.<br>Check for early warning signs.<br>Manage triggers with lifestyle skills.
-2 Mild Down	Take medications daily.<br>Use lifestyle skills.<br>Use coping skills.<br>Use supports. Work more closely with your psychiatrist.
-3 Moderate Down	Take medications daily. Use lifestyle and coping skills.<br>Use supports.<br>Call your psychiatrist.<br>Consider more intensive treatment.
-4 Severe Down	Call your psychiatrist.<br>Call 911.<br>Go to the hospital.

My Team

My Doctor

Name
(xxx) xxx-xxxx

Other Mental Health Providers

Name
(xxx) xxx-xxxx

My Pharmacy

Name
(xxx) xxx-xxxx

My Hospital

Name
(xxx) xxx-xxxx

My Family

Name
(xxx) xxx-xxxx

My Friends

Name
(xxx) xxx-xxxx

My Skills

- I. Fundamentals of Skills +
- II. Lifestyle Skills -
  - A. Blueprint +
  - B. Abstinence +
  - C. Sleep -
    - Three day challenge >
    - Clean castle >
    - Worry pad >
    - Worry chair >
    - Imagery >
  - D. Interactions +
  - E. Calm +
- III. Coping Skills +

Settings & Instructions

I. Introduction +

II. Schedule +

III. Reminders +

IV. Settings +

V. Coach +

VI. Psychiatrist +

VII. Foundations +

VIII. Check Ins -

A. Daily -

Wellness rating >

Early warning signs >

Medications >

Sleep >

B. Weekly +

IX. Daily Review +

X. Charts +

XI. Wellness Plan +

XII. FAQs +
