# Supplementary material for: A Smartphone-Based Self-management Intervention for Bipolar Disorder (LiveWell): User-Centered Development Approach
Source: JMIR Ment Health. 2021 Apr 12;8(4):e20424. doi: 10.2196/20424 (PMC8076988; doi:10.2196/20424)
Supplement: Multimedia Appendix 4 [file mental_v8i4e20424_app4.pdf]

ID: ________________________Date: _______________________ Interviewer: ___________________

Introduction

Hi, __________. My name is __________, and I'm going to be walking you through this session today. Before we begin, I have some information for you, and I'm going to read it to make sure that I cover everything.

You probably already have a good idea of why we asked you here, but let me go over it again briefly. We're asking people to take part in a usability test of a prototype phone application for people with bipolar disorder. We need to do some testing to determine how well our design works and whether it works as intended. The session should take an hour.

As a volunteer in this study, your participation will be anonymous. If for any reason you are uncomfortable during the session, you can choose to stop your participation at any time.

Usability Test Description

During this session, I will be asking you to complete a few common tasks using the application on a study smart phone. The first thing I want to make clear right away is that we're testing the *application,* not you.

I'd like you to complete the tasks as if you were in your home. I'm going to ask you as much as possible to think out loud: to say what you're looking at, what you're trying to do, and what you're thinking. This will be a big help to us.

Also, please don't worry that you're going to hurt our feelings. We're doing this to improve the application, so we need to hear your honest reactions.

If you have any questions as we go along, just ask them. I may not be able to answer them right away, since we're interested in how people do when they don't have someone sitting next to them to help. But if you still have any questions when we're done I'll try to answer them then. And if you need to take a break at any point, just let me know.

As you use the application, I will be watching and taking notes. You may have noticed a camera in the corner of the room. With your permission, we're going to record what happens during this session and our conversation. The recording will only be used to help us figured out how to improve the application, and it won't be seen by anyone except the people working on this project.

Do you have any questions before we begin?

____

____

____

ID: _________________________Date: ________________________ Interviewer: ____________________

Application Overview

Take a few minutes to look over the application. Please share any thoughts you have out loud as you go along and ask any questions that come to mind.

[Allow 3-5 minutes for participant to look over the application.]

Comments:____

____

____

____

What is your overall impression?

____

____

____

Anything in particular you liked?

____

____

____

Anything in particular you didn’t like?

____

____

____

ID: ________________________Date: _______________________ Interviewer: ___________________

Foundations

Now I'm going to ask you to read two different foundation lessons. I will select the first lesson for you to read and the second lesson will be your choice.

I'm going to read the name of the first lesson out loud. I'm going to ask you to find this lesson on your phone and read it. I will say START to signal that you can begin.

Assigned lesson #1: ___________________________

Time to complete task: _________________

Observations: ____

____

____

Any thoughts about the lesson?

____

____

____

How useful do you think the content was?

____

____

____

What did you think about the length of the lesson?

____

____

____

Was there anything that wasn't covered that you think would be useful?

____

____

____

ID: ________________________Date: _______________________ Interviewer: ___________________

Could you name at least one thing you liked?

____

____

____

Could you name at least one thing you didn't like?

____

____

____

Great. The second lesson you will read is your choice. I would like you to select a Foundations lesson that interests you. When you have selected a lesson, state your choice out loud. I will then say START to signal that you can begin reading that lesson.

Participant's choice #2: ____________________________

Time to complete task: _________________

Observations: ____

____

____

Any thoughts about the lesson?

____

____

____

What did you think about the length of the lesson?

____

____

____

ID: _________________________Date: ________________________ Interviewer: ___________________

How useful do you think the content was?

____

____

____

Was there anything that wasn't covered that you think would be useful?

____

____

____

Could you name at least one thing you liked?

____

____

____

Could you name at least one thing you didn't like?

____

____

____

<u>Scenarios</u>

Now I'm going to ask you to try doing some specific tasks. I'm going to read each one out loud and give you a printed copy. After reading the task, I will say START to signal that you can begin.

And again, as much as possible it will help us if you can try to think out loud as you go along.

ID: ________________________ Date: _______________________ Interviewer: ___________________

SCENARIO #1: WELL

Time to complete task: ________________ ☐ Task not completed

Comments:____

____

____

How do you feel about getting this feedback?

____

____

____

How useful do you think this feedback was?

____

____

____

Would something else be more useful?

____

____

____

Could you name at least one thing you liked?

____

____

____

Could you name at least one thing you didn't like?

____

____

____

ID: ________________________ Date: _______________________ Interviewer: ___________________

SCENARIO #2: MEDICATIONS

Time to complete task: ________________ ❑ Task not completed

Observations:____

____

____

How do you feel about getting this feedback?

____

____

____

How useful do you think this feedback was?

____

____

____

Would something else be more useful?

____

____

____

Could you name at least one thing you liked?

____

____

____

Could you name at least one thing you didn’t like?

____

____

____

ID: ________________________Date: _______________________ Interviewer: ___________________

SCENARIO #3: SLEEP LESS

Time to complete task: _______________ ❑ Task not completed

Observations:____

____

____

How do you feel about getting this feedback?

____

____

____

How useful do you think this feedback was?

____

____

____

Would something else be more useful?

____

____

____

Could you name at least one thing you liked?

____

____

____

Could you name at least one thing you didn’t like?

____

____

____

ID: ________________________ Date: _______________________ Interviewer: ___________________

SCENARIO #4: ROUTINE

Time to complete task: ________________ ❑ Task not completed

Observations:____

____

____

How do you feel about getting this feedback?

____

____

____

How useful do you think this feedback was?

____

____

____

Would something else be more useful?

____

____

____

Could you name at least one thing you liked?

____

____

____

Could you name at least one thing you didn’t like?

____

____

____

ID: ______________________Date: _____________________ Interviewer: _________________

SCENARIO #5: PRODROME DOWN

Time to complete task: _______________ ❑ Task not completed

Observations:____

____

____

How do you feel about getting this feedback?

____

____

____

How useful do you think this feedback was?

____

____

____

Would something else be more useful?

____

____

____

Could you name at least one thing you liked?

____

____

____

Could you name at least one thing you didn’t like?

____

____

____

ID: _________________________Date: ________________________ Interviewer: ___________________

[Administer post-task questionnaire]

Thank you

Interviewer Copy

Task Scenario 1 – Doing well

Imagine that you have been doing well for several weeks. You have been taking all of your medications every day, getting adequate sleep, and keeping a regular routine. You have been rating yourself as doing well.

You are continuing to do well. Go ahead and check in.

Participant Copy

Task Scenario 1 – Doing well

Imagine that you have been doing well for several weeks. You have been taking all of your medications every day, getting adequate sleep, and keeping a regular routine. You have been rating yourself as doing well.

You are continuing to do well. Go ahead and check in.

Interviewer Copy

Task Scenario 2 – Taking medications

Imagine that you have been doing well for several weeks. You have been getting adequate sleep and keeping a regular routine. However, for the past couple of days you have forgotten to take your evening medications. Yesterday you forgot to take both your morning and evening medications.

Go ahead and check in.

Participant Copy

Task Scenario 2 – Taking medications

Imagine that you have been doing well for several weeks. You have been getting adequate sleep and keeping a regular routine. However, for the past couple of days you have forgotten to take your evening medications. Yesterday you forgot to take both your morning and evening medications.

Go ahead and check in.

Interviewer Copy

Task Scenario 3 – Sleeping too little

Imagine that you have been doing well for several weeks. You have been taking your medications every day and keeping a regular routine. However, for the past couple of nights you have been sleeping less than usual, sometimes as much as 2 hours less. Last night you had a particularly tough time getting to bed and only got 5 hours of sleep.

Go ahead and check in.

Participant Copy

Task Scenario 3 – Sleeping too little

Imagine that you have been doing well for several weeks. You have been taking your medications every day and keeping a regular routine. However, for the past couple of nights you have been sleeping less than usual, sometimes as much as 2 hours less. Last night you had a particularly tough time getting to bed and only got 5 hours of sleep.

Go ahead and check in.

Interviewer Copy

Task Scenario 4 – Maintaining a routine

Imagine that you have been doing well for several weeks. You have been taking your medications every day and getting adequate sleep. However, your routine has been off this past week. Your boss recently assigned you to a later shift at work and this has interfered with your normal routine. Last night you went to bed much later than usual and slept well into the afternoon the next day.

Go ahead and check in.

Participant Copy

Task Scenario 4 – Maintaining a routine

Imagine that you have been doing well for several weeks. You have been taking your medications every day and getting adequate sleep. However, your routine has been off this past week. Your boss recently assigned you to a later shift at work and this has interfered with your normal routine. Last night you went to bed much later than usual and slept well into the afternoon the next day.

Go ahead and check in.

Interviewer Copy

Task Scenario 5 – Early warning signs of depression

Imagine you have generally been doing well for the past couple of days. You have been taking your medications every day, and your mood and energy levels have been good. However, yesterday you started feeling a little bit down, maybe even a little depressed. At first you shrug it off as just a case of the blues, but upon closer inspection, you think you may be experiencing early warning signs of depression. Last night you didn't sleep well at all. It took you longer than usual to fall asleep, and you woke up very early this morning and weren't able to go back to sleep. You are feeling sluggish and are having trouble getting motivated to start your day.

Go ahead and check in.

Participant Copy

Task Scenario 5 – Early warning signs of depression

Imagine you have generally been doing well for the past couple of days. You have been taking your medications every day, and your mood and energy levels have been good. However, yesterday you started feeling a little bit down, maybe even a little depressed. At first you shrug it off as just a case of the blues, but upon closer inspection, you think you may be experiencing early warning signs of depression. Last night you didn't sleep well at all. It took you longer than usual to fall asleep, and you woke up very early this morning and weren't able to go back to sleep. You are feeling sluggish and are having trouble getting motivated to start your day.

Go ahead and check in.
