# Supplementary material for: A Smartphone-Based Self-management Intervention for Bipolar Disorder (LiveWell): User-Centered Development Approach
Source: JMIR Ment Health. 2021 Apr 12;8(4):e20424. doi: 10.2196/20424 (PMC8076988; doi:10.2196/20424)
Supplement: Multimedia Appendix 6 [file mental_v8i4e20424_app6.pdf]

LiveWell: Pilot Study Application Training

Study ID:________________ Training Date:____________ Trainer:__________________

**Prepare for the participant**
• Equipment: pre-loaded phone/charger, watch/charger, audio recorder, $<br>• Summary sheet from assessor<br>• Participant handouts

1. Introduction

Time: _________

Welcome. Thanks for coming to participate in the *LiveWell* study.

I'm [name]. I will be working as a coach to follow along with you while you participate in the *LiveWell* study.

- As we talk today, I'll take some notes
- Audio recording- only study team listens
  - Use to improve how we help people use the application
- Today, about 1 hour
- Go over how to use the equipment
- Go over how to use the self-management application
- May seem like a lot so don't worry about every detail
- Everything we cover today is in Instructions section of app
- You can call or email me with questions as well

Do you have any questions right now?

____

____

____

____

____

____

____

____

Study ID:________________ Training Date:____________ Trainer:__________________

2. Hopes

[Use participant responses to emphasize how app will be useful to them.]
[Build rapport. Support self-efficacy.]

How did you decide to participate in this program?

____

____

____

____

What would you like to be different at the end of this program?

____

____

____

____

3. Overview

LiveWell focuses on self-management tools to help decrease mood episodes.

- Medications shown reduce risk symptoms and episodes in bipolar disorder
- <u>Most</u> people know about the need for medications in bipolar disorder
- Self-management cuts down symptoms up to 50% compared with medications alone
- <u>Many</u> people do not know about these tools

What do you do to manage bipolar disorder now?

[Support participant's efforts to stay well.] [Make links to how *LiveWell* may be useful if possible.]

____

____

____

____

Study ID:________________ Training Date:____________ Trainer:__________________

General Rationale and Targets

LiveWell: learn about and practice strategies that focus on ways to:

1. Take medications as prescribed
2. Get the right amount of sleep
3. Keep a regular routine
4. Notice early signs or symptoms and take action to decrease the chance of an episode

Time-Limited

The application is designed to be used in a time-limited fashion over a 2 month period:

- Help you learn more about your symptoms
- Identify ways to reduce their likelihood of occurring
- Manage them when they occur

Hopefully, after using *LiveWell* for 2 months you will be able to better manage bipolar disorder without needing to use the application daily.

4. Wellness Rating Scale Time: _________

- Important part of what you will be doing with *LiveWell* is checking in daily
- Involves determining where you are in terms of your personal wellness
- Different people describe this differently
- So before we go to the application, let's create your personalized wellness rating scale

[Give out handout 1: Fillable Anchors, show 9 ratings and standard definitions]

- Will use 9 wellness ratings to check in and help you rate how you are doing each day
- You will write down 3-5 brief statements on handout for each rating
- Can describe your
  - Moods and emotions
  - Kind of thoughts you have
  - Whether your thinking is fast or slow
  - Your behaviors, sleep, or energy levels
  - Your interests or outlook on life
  - How you relate to others
- I will load anchoring statements on the phone for you so best to keep reminders brief
- Will review in 4 weeks, can update for you if you want to change at anytime
- Objective of this is to create personal, meaningful reminders for well, depressed, manic, in between

LiveWell: Pilot Study Application Training

Study ID:________________ Training Date:____________ Trainer:__________________

Wellness Scale (-3)

On the rating scale, episodes of depression correspond to feeling **moderately down (-3)**.

With this type of down people usually have:

- Multiple symptoms
- Symptoms that continue day after day
- May have difficulty maintaining their usual activities and routines.

Can you write down a few words or phrases that describe you when you are having a depressive episode?

Ok. Now let's take a look at this handout that covers the main symptoms of depression.

[Give out handout 2: Symptoms of Depression.]

Are there any symptoms on this list that resonate more with you? If so, adjust your sheet accordingly.

So what did you write down?

[If describing most severe depression or crisis level of depression, anchor as -4, then return to -3]

That will be moderate down, a rating of -3, consistent with a depressive episode.

Coach notes:

____

____

____

____

Wellness Scale (-4)

Let's consider what would be a crisis situation due to depression. This might include:

- Thinking about suicide including making or acting on plans to kill yourself
- Experiencing psychotic symptoms such as delusions or hallucinations
- Being unable to maintain your regular activities, responsibilities, and routines
- Being unable to sleep, eat, or bath regularly
- Engaging in behaviors with serious consequences (risky, dangerous)

Study ID:________________ Training Date:____________ Trainer:__________________

This would be a depressive crisis or **severe down (-4)**.
[If past episodes of suicidality, hospitalization due to depression, consider what that was like.]
[If never suicidal, hospitalized or very severe depression, consider what would lead to 911/hospital.]

Can you write down a few words or phrases that would describe you at such a time?

So what did you write down? [Make sure participant is on track]

This will be severe down, a rating of -4, which means a crisis due to severe depression.

Coach notes:

____

____

____

Wellness Scale (-2)

On the rating scale, early warning signs of depression correspond to feeling **mildly down (-2)**.

- Many people notice low level symptoms or other signs before a depressive episode
- These changes happen first and often predict an episode of depression
- These kinds of changes are called early warning signs
- Learning to notice and take action helps avoid episodes

People often maintain their usual activities and routines when early warning signs are present.

Write a few words or phrases that describe how you are before an episode of depression. Try to list signs or symptoms that come FIRST for you.

[Give out and look over handout 3: Early Warning Signs of Depression]

Here is a list of early warning signs of depression that are pretty common. Are there any on this list that resonate with you that you'd like to add to your sheet?

So what did you write down? [Make sure participant is on track]

This will be mild down, a rating of -2, which means early warning signs of depression.

Coach notes:

____

____

____

Study ID:________________ Training Date:____________ Trainer:__________________

<u>Wellness Scale (+3)</u>

On the rating scale, episodes of mania correspond to feeling **moderately up (+3)**.

With this type of up people usually have:

- Multiple symptoms
- Symptoms that continue day after day
- May have difficulty maintaining their usual activities and routines.

Can you write some words or phrases that describe when you are having a manic episode?

Ok. Now let's take a look at this hand out that covers the main symptoms of mania.

[Give out and look over handout 4: Symptoms of Mania.]

Are there any symptoms on this list that resonate more with you? If so, adjust your sheet accordingly.

So what did you write down? [If describing most severe mania or crisis level of mania, anchor as +4, then return to +3]

This will be moderate up, a rating of +3, consistent with a manic episode.

Coach notes:

____

____

____

____

<u>Wellness Scale (+4)</u>

Let's consider what would constitute a crisis situation due to a manic episode.

This might include:

- Being unable engage in your daily activities, responsibilities, and routines
- Being unable to sleep, eat or bath regularly
- Engaging in behaviors with serious consequences (risky, dangerous, fighting, promiscuous, spending)
- Having very poor judgment, making very bad decisions
- Experiencing psychotic symptoms such as delusions or hallucinations

Study ID:________________ Training Date:____________ Trainer:__________________

This would be a manic crisis or **severe up (+4)**.

[If past episodes of severe mania, hospitalization due to mania, consider what that was like]

[If never hospitalized or very severe mania, consider what lead to 911/hospital]

Can you write down a few words or phrases that would describe you at such a time?

So what did you write down? [Make sure participant is on track]

This will be severe up, a rating of +4, which means a crisis due to severe mania.

Coach notes:

____

____

____

____

<u>Wellness Scale (+2)</u>

On the rating scale, early warning signs of mania correspond to feeling **mildly up (+2)**.

- Many people notice low level symptoms or other signs before entering a manic episode
- These changes happen first and often predict an episode of mania
- These kinds of changes are called early warning signs
- Learning to notice and take action helps avoid episodes

People often maintain their usual activities and routines when early warning signs are present.

Write a few words or phrases down that describe this how you are before an episode of mania. Try to list signs or symptoms that come <u>FIRST</u> for you.

Here is a list of early warning signs of mania that are pretty common. Are there any on this list that resonate with you that you'd like to add to your sheet?

[Give out and look over handout 5: Early Warning Signs of Mania]

So what did you write down? [Make sure participant is on track]

This will be mild up, a rating of +2, which means early warning signs of mania.

Study ID:________________ Training Date:____________ Trainer:__________________

Coach notes:

____

____

____

____

<u>Wellness Scale (0)</u>

Let's cover when you are doing **well** and feeling **balanced (0)**.

Write down a few words or phrases that describe what it's like for you when you are balanced.

Think about:

- Your mood
- Your thoughts
- Your behaviors
- Your personality
- Your interests
- Your outlook on life
- How you relate to others

So what did you write down? [Make sure participant is on track]

This will be a 0.

Coach notes:

____

____

____

____

Study ID:________________ Training Date:____________ Trainer:__________________

Wellness Scale (-1)

Next we can talk about times when you feel **slightly down (-1)**.

This type of down is:

- Typical response to routine negative event in daily life
- Usually a normal variation in mood, thoughts, or behavior

Write down a few words or phrases that you at these times.

So what did you write down? [Make sure participant is on track]

This will be a -1.

Coach notes:

____

____

____

____

Wellness Scale (+1)

Now think about times when you feel **slightly up (+1)**. This type of up is:

- Typical response to routine positive event in daily life
- Usually normal variation in mood.

Write down a few words or phrases that you at these times.

So what did you write down? [Make sure participant is on track]

This will be a +1.

Coach notes:

____

____

____

____

Study ID:________________ Training Date:____________ Trainer:__________________

5. Home Page

Now let's start going over the application.

- Let me know if going too fast/slow
- Ask questions any time

[Give participant the phone. Show where to go on the phone for application icon.]
[Probe for Android/smart phone experience. How comfortable do you feel?]

Let's look at the home page of the *LiveWell* application. You will see this home page when you open the application. Right now, there are a few buttons on the home page:

- Weekly Check In
- Daily Check In
- Foundations
- Toolbox
- Wellness Plan

An **important part of *LiveWell* is checking in and reviewing** how you are doing every day. White tabs indicate tasks that *LiveWell* is asking you to complete today.

There are three main parts other than the daily and weekly check ins:

- Foundations
- Toolbox
- Wellness Plan

Over next 4 weeks, you will be reading the Foundations, becoming familiar with the Toolbox, and building your own Wellness Plan.
I'll work with you each week during the first month going through it all step-by-step with you.

6. Check Ins

Time:_________

Check Ins: Prompts

Every day application displays a reminder to check in:

- Comes up in tray [Show where will be]
- Will be there in tray until complete *LiveWell* activities for the day

Check Ins: Settings [Make sure patient on home page and show where settings button is]

Set when would like to receive first reminder of the day to complete *LiveWell* activities:

- Daily Check In
- Daily Review
- Weekly Check In (on Sundays only)

Study ID:________________ Training Date:____________ Trainer:__________________

If not a good time or don't complete daily check-in:

- Alert icon will stay in tray
- You will get three reminder tone or buzzes from phone: one at time reminder is scheduled, then 2 and 4 hours after that
- If you find the reminder is routinely arriving at a bad time, feel free to change the settings to fit your schedule.
    - [Check in time should be 8pm or earlier for logistical reasons]

Any questions about setting when you want to check in or about the reminders?

____

____

____

7. <u>Daily Check Ins</u>

Let's go over each part of the daily check in.

[Have participant navigate to the Daily Check In page, Review Daily Check In areas and why complete]

<u>Check Ins: Daily – Medications</u>

- To reduce symptoms and episodes important take medications
- Can be difficult for people to take medications daily

- Under medications in Daily Check In, 3 buttons: All, Some and None
- Record took all, some, or none daily psychiatric medications last day by push button
- Do not need to include PRN medications or medications for other conditions

What is your target for taking your medications for bipolar disorder? (percent adherence)
[Attempt to engage for all]

____

____

____

____

Study ID:________________ Training Date:____________ Trainer:__________________

Check Ins: Daily – Sleep

To reduce symptoms and episodes important to get the right amount of sleep.

- General recommendation by the American Sleep Foundation is 7-9 hours a night with 6-10 hours being ok for some
- Keeping track of your sleep can be useful for trying to get recommended hours sleep
- Changes in sleep duration can be a sign of an upcoming episode

- Enter the number of hours you think you slept each night [not time in bed]
- Don't include naps.

[Go over how use slider to input sleep duration in half hour increments]

What is your target for amount of sleep?

[Attempt to engage for a two hour window that remains between 6 to 10 hours]

____

____

____

____

Check Ins: Daily – Routine

To reduce symptoms and episodes also important to keep a regular schedule. In particular, try to do these 5 activities at about the same time every day:

- Get out of bed
- First have contact with another person
- Start work/school/volunteer/family care
- Dinner
- Get to bed

Daily check in asks you to keep track of when you go to bed for the night/get up for the day

- Push button to record when to bed the night before with the intention of going to sleep
- Selected time, not when you went to sleep but when you tried to get to sleep
- Push button to record when you got up planning to start your day
- Select time you got up
- Be mindful of AM/PM for times selected

Study ID:________________ Training Date:____________ Trainer:__________________

What is your target for routine?

[Attempt to engage for consistency; 1.5 hour window for bedtime and rise-time]

____

____

____

____

Check Ins: Daily – Wellness Scale

To reduce symptoms and episodes important to be aware of how you are doing and taking action if early warning signs appear. Can also help to manage any residual mood symptoms.

Each day record wellness rating (for the previous day) by pressing the rating on daily check in:

- Remember review in your mind how you did over the entire prior day (or past 24 hours)
- Don't rate how you are doing just in the moment you are checking in
- Take a moment to reflect an how you are doing overall
- Think about the anchors for the scale that we have created
- Review your anchors and general definitions if you aren't sure how you did
- Once you press the number, turns white, so you know it has been selected

What is your target for monitoring and managing early warning signs and symptoms?

[Attempt to engage for monitoring ews]

____

____

____

____

[Have participant return to the home page when prompt for daily review comes up]

Study ID:________________ Training Date:____________ Trainer:__________________

8. Daily Review

[Help participant navigate to the Daily Review]

Like the Daily Check In, we recommend you complete the Daily Review every day:
- Comes up automatically after Daily Check In
- Can complete right after Daily Check In or later if not enough time

The Daily Review bar graphs summarize last 7 days how doing with and provides feedback:
- taking Medications
- getting Sleep
- maintaining regular Routines
- your Wellness

How it works:
- Based on your Daily Check Ins for current day and last 6 days checked in
- Percentage of success for each target (e.g. 100% of meds)

[Show hover bars, cover how % works, set personal target sleep duration, bed/rise-time later]

- If all well, option learning more about skills/tools for living well
- If something amiss,
    - Provides options to explore solutions
    - May suggest call psychiatrist
- Prompt you back to relevant wellness plan, foundations, and toolbox
- After complete review, can repeat suggested Daily Review from home screen
- Also can “Review Something Else” of your choice
    - From end of the daily review
    - From the home screen

[Have participant complete the Daily Review]

How might you find the daily check in and daily review useful? Questions?

____

____

____

____

Study ID:________________ Training Date:____________ Trainer:__________________

9. Weekly Check Ins Time: _________

[Have participant navigate to the Weekly Check In]

Weekly Check In:

- Comes up on Sundays
- 4 sections

First two sections:

- Questions about depression and mania
- Please enter an answer for each question
- App will prompt you to enter an answer if you don't
- Your responses should consider the entire past week, not just the day of check in

Last two sections:

- Checklists for early warning signs of depression and mania
- Check off any that you noticed in the last week
- Remember these should be early warning signs of a mood episode (+/- 2)
- Not just slight ups and downs that you might expect in response to events (+/- 1)
- If you didn't experience any early warning signs then just leave the checklist blank

[Have participant complete weekly check in]

10. Clinical Status Summary

You will have access to an online dashboard that displays an overview of how you are doing. I will email you a link and password in separate emails. The reports do not contain your name or other identifiers, and the website is secure.

[Handout 6 Clinical Status Summary or Navigate to: https://mohrlab.northwestern.edu/livewell-dash/, describe information presented]

You can allow your psychiatrist or other mental health provider to review this overview report of how you are doing.

[Ask if want to give anyone access to the reports]
[If yes, get name and email and complete authorization]

Your mental healthcare provider must opt in to have access to the dashboard. If they would like to see the reports, they will receive a link and password separately to ensure security. You can withdraw their access to the dashboard at any time.

Are you comfortable with your provider having access to this information? [Reassure that dashboard is secure, remind that no names used on actual reports.]

Study ID:________________ Training Date:____________ Trainer:__________________

Based on Daily Check In, Daily Review may prompt you to call your psychiatrist if problems:
- Taking medications
- Getting adequate sleep
- Early warning signs or symptoms
- Severe symptoms

We will always contact you if it appears you are not doing well based on your self-report data. We may also contact your psychiatrist. However, since we are only here M-F 9-5, you should contact your psychiatrist right away if you are having severe symptoms consistent with a crisis.

It is essential that you call your psychiatrist if you are having problems.

If your psychiatrist opts in, the application may also send email notifications to your psychiatrist. Any time your psychiatrist receives an email alert or alert call from me, the application will also recommend you call your psychiatrist. Your psychiatrist may not:
- Check the summary reports on the dashboard regularly
- Respond promptly to alert calls or email alerts
- **Contact them directly if you are having symptoms or other problems**

Do you have any questions about the *LiveWell* reports and notification?

____

____

____

____

11. Instructions

[Show participant that Instructions are available from the home page]
- Covered a lot today
- Instructions section goes over what talked about today

12. Refresh

[Show participant the refresh button on Home Page]
- Refresh button allows content in the application to update
- You can update anytime by pressing the Refresh button
- If content seems old or is inconsistent with most recent conversation with me, we recommend pressing refresh button before letting me know
- Push the Refresh button tomorrow to update based on information you gave me today

Study ID:________________ Training Date:____________ Trainer:__________________

13. Summarize

What it's designed to do:

- App - help you learn how to better manage bipolar disorder
- Goal - reduce likelihood of symptoms, better manage symptoms when they occur

Important clarifications:

- Not a replacement for working with your psychiatrist and other mental health providers
- Not an emergency service
- As your coach, help you get most out of app but not a therapist
- Urgent psychiatric problem, contact your psychiatrist immediately
- Although app may provide summary information for psychiatrist, not a replacement for talking with your psychiatrist about:
  - Symptoms consistent with an episode
  - Early warning signs that you cannot reduce with self-management in 1-3 days
  - Any ongoing mental health or medication problems you are having

If you are feeling suicidal or getting into dangerous situations, you should:

- Call 911
- Go to the nearest emergency room

14. Equipment

You might be aware that activity tracking devices are a new trend to help live a healthy lifestyle and maintain a routine. Your study phone and watch will collect behavioral data. We are examining how this information may be useful for helping those with bipolar disorder.

We ask that you keep both the phone and the watch on and fully charged, and that you wear the watch and carry the phone at all times.

The phone has an application installed, called Purple Robot, that collects data about activity, location, calls, and texts (which does not include information about content or identities)

The pebble watch sends feedback from its sensors to the phone to track sleep, activity level

- Apologize if it feels bulky on wrist. So appreciative if you can wear it 24/7
- Water resistant
- Prefer worn on non-dominant wrist

If you are experiencing any technological issues, you can call or email me anytime.

[Make sure that seems feasible. Make relationship to result in meaningful data]

Study ID:________________ Training Date:____________ Trainer:__________________

15. <u>Coach's Role</u> Time: __________

- Help learn how to use the application so most helpful for you
- Set up phone calls to see how things are going
- Opportunity for you to ask questions about application
- Can also contact me by email at LiveWellCoach@Northwestern.edu
- Phone at 312-503-1886
- This info is in Instructions → Coach

During the first month, work with you to go over basic ideas about managing bipolar disorder.

This information is in Foundations. There are 8 Foundations modules:

- Each module takes about 5-10 minutes to read
- Read all the modules during the 1st month of the program
- About two modules a week 1st month

<u>For the first 3 weeks</u>, we'll schedule a brief 15 min phone call each week and review:

- The modules for that week
- How you have been using the application
- Any difficulties with the application
- Goals for using the application and meeting your targets

<u>During the fourth week</u>, we'll have a longer 30-40 minute call to:

- Wrap up last modules
- Build your personal wellness plan.
- <u>We will review this plan two weeks later, during week six</u>

You can also call anytime if having problems with using application, phone, or watch.

<u>After week 6,</u> we'll schedule a call to discuss wrapping up your daily use of the application, to talk about your experience of the LiveWell program, and your long terms plans for managing bipolar disorder.

Prior to scheduled calls, I will review your application use and will have a sense of how things are going. This includes your daily check-in data and whether or not you took a look at the foundations.

I'll also call you, if it appears you are not using checking in daily.

Study ID:________________ Training Date:____________ Trainer:__________________

What do you think about these coaching calls?
[Work toward mutual expectations for use of application and completion of lessons.]
[Supportive accountability. Address privacy concerns.]

____

____

____

How about this schedule?
[Elicit commitment. Find out about potential obstacles.]
____

____

____

____

16. <u>Commitment and Goals</u>

[Summarize participant's story, dilemmas/strengths, barriers/solutions]
[Highlight "change talk" and perceived positives of *LiveWell* program. Link to hopes.]

To get started we recommend that you:

- Read 2 modules a week during the 1st month
- Use the daily check in and daily review every day for 2 months
- Use the application to assist with
    - Taking medications daily
    - Getting the right amount of sleep
    - Keeping a regular routine
    - Watching for signs that might indicate you are heading toward an episode

[Summarize targets]

Study ID:________________ Training Date:____________ Trainer:__________________

What do you think about your targets?

[Explore think useful achieve targets and if achievable. Provide meaningful rationales as needed.]
[Acknowledge negative feelings about requesting attempt meet target behaviors.]
[Use non-controlling language (avoid should, must, have to).]
[Problem solve. Be flexible. Offer choices -how might use application to help, what focus on first.]
[Elicit commitment emphasize competence, curiosity, and interest.]

____

____

____

____

Do you think using the application might help you achieve these targets? How?

____

____

____

____

17. Program Participation Obstacles and Problem Solving

You said you were hoping for [name hopes]. **Now that you know more about application, think *LiveWell* program help you get there? Exactly how?**

____

____

____

____

Study ID:_________________ Training Date:_____________ Trainer:___________________

Beyond concerns talked about [name them], **what might make hard to participate in *LiveWell*?**

____

____

____

____

What might you or we do get past these obstacles so can benefit most from *LiveWell*?

____

____

____

____

We recommend daily use of the *LiveWell* application. What do you think about that?

____

____

____

____

18. Wrap Up

Okay, let’s talk about the week ahead. We recommend you:

- Read the Overview and Basic Facts in foundations before we talk next week
- Use the daily check in and daily review every day

How does the goal of doing this for the next week sound to you?

____

____

____

Study ID:________________ Training Date:____________ Trainer:__________________

Do you have any other goals in terms of using the application for the next week?

____

____

____

____

Ok, so we will talk next week. **Set up a time** ________________

- I appreciate your taking such an active part in learning about the program today.
- I really think you will find this program useful in helping you stay well.
- Sometimes things come up and people aren't able to use the program as planned.
- We can always talk about any problems and I'm happy to help.

Do you have any questions for me?

____

____

____

____

Thank you for coming in today.

Summary:

____

____

____

____

____

____

Time:__________

REMEMBER: Administer RedCap PDQ and pay participant

Wellness Rating Scale

Wellness	Definition	Anchors
**+4 Severe Up**	Poor judgement. Dangerous behaviors Not sleeping. Hallucinations/delusions.	
**+3 Moderate Up**	Many symptoms day to day. Manic episode probably happening. Difficult to maintain activities/routine.	
**+2 Mild Up**	Some symptoms, early warning signs. Manic episode may be coming. Can still maintain activities/routine.	
**+1 Slight Up**	Response to recent/upcoming good event. Likely normal variation in wellness. Understandable and manageable.	
**0 Balanced**	Neither up nor down. Doing well.	
**-1 Slight Down**	Response to recent/upcoming bad event. Likely normal variation in wellness. Understandable and manageable.	
**-2 Mild Down**	Some symptoms, early warning signs. Depressive episode may be coming. Can still maintain activities/routine.	
**-3 Moderate Down**	Many symptoms day to day. Depressive episode probably happening. Difficult to maintain activities/routine.	
**-4 Severe Down**	Serious ideas about suicide. Immobilized. Dangerous behaviors. Disrupted sleep. Hallucinations/delusions.	

Symptoms of Depression

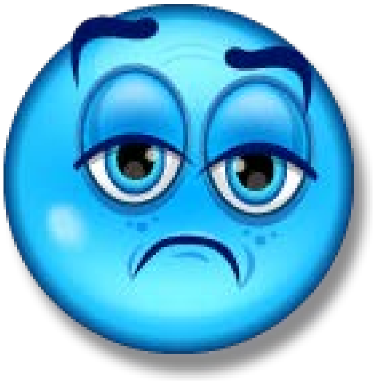

The hallmark of depression is **low mood or diminished interest and pleasure in life**. Other symptoms include:

- ✓ Weight loss or weight gain
- ✓ Sleeping too much or too little
- ✓ Physical agitation or slowing down
- ✓ Fatigue or loss of energy
- ✓ Feeling worthless or guilty
- ✓ Difficulty concentrating
- ✓ Thoughts of death or suicide

Low mood or diminished interest/pleasure plus 4 or more other symptoms on **most days for 2 weeks** is considered a full-blown depressive episode.

Early Warning Signs of Depression

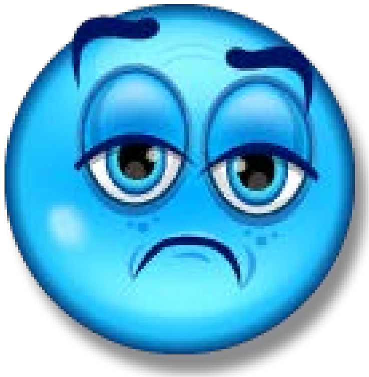

Many people notice low level symptoms or other signs well before entering a depressive episode. These are called early warning signs. Common early warning signs include:

- ✓ Sad or anxious mood
- ✓ Less energy than usual
- ✓ Problems concentrating
- ✓ Less interest than usual
- ✓ Negative thinking
- ✓ Withdrawn
- ✓ Sleep disturbance
- ✓ Guilt

How can you tell if an episode of depression is coming? What changes do you recognize in yourself that lead up to a depressive episode? Are there any signs that you notice **first**?

Symptoms of Mania

The hallmark of mania is a **euphoric or irritable mood**. Other symptoms include:

- ✓ Inflated self-esteem/grandiosity
- ✓ Decreased need for sleep
- ✓ More talkative than usual
- ✓ Racing thoughts
- ✓ Difficulties concentrating
- ✓ Increased activity level
- ✓ Risky activities

Elevated or irritable mood plus 3 or more other symptoms **for a week** is considered a full-blown manic <u>episode</u>.

Early Warning Signs of Mania

Many people notice low level symptoms or other signs well before entering a manic episode. These are called early warning signs. Common early warning signs include:

- ✓ Sleep disturbance
- ✓ More active than usual
- ✓ More talkative than usual
- ✓ More social than usual
- ✓ More irritable/agitated than usual
- ✓ Increased energy
- ✓ Increased self-esteem
- ✓ Racing thoughts

How can you tell if an episode of mania is coming? What changes do you recognize in yourself that lead up to a manic episode? Are there any signs that you notice **<u>first</u>**?

LiveWell: Scheduled Coaching Calls Pilot Study FV

Study ID:__________ Coach:__________ Date of session:__________

WEEK 1

Get prepared with data since last call…

Medications:	Sleep:	Routine:	Wellness Ratings:	Use of App (check ins):
Weekly Goals (training session):				
Hopes (Initial session):				

Coach only:

Daily Review feedback category:	LiveWell Clinical Status:	Weekly Survey Scores	
		PHQ8	ASRM

1. <u>Aims</u>

Hi [participant]. This is your coach, [name], from the *LiveWell* program. I'm calling for our Week 1 check in. It should take about 15 minutes. Is that alright?

Today, we are scheduled to review 2 lessons (Overview and Basic Facts), how using the daily check in and daily review went, and your goals from the phone training.

Is there anything else you would like to cover?

____

____

____

Any problems, questions, or concerns about using the application? Does your personalized content look up to date [anchors, meds, psych info]?

____

____

____

____

Study ID:__________ Coach:__________ Date of session:__________

2. <u>Review</u>

Let's talk about how things are going. Based on the data I have received, it looks like… [summarize dashboard data including wellness ratings, symptoms, targets, use of application].

Does that sound right?

____
____
____
____

[Support awareness, action, and success with targets. Explore problems with targets, ews monitoring, and implementing plan.]

[Only revise targets after repeated failures unless good reason to do so (i.e. started new job and schedule has shifted).]

<u>Goals</u>

Last time you hoped [initial session goal]. To what extent were you able to achieve this goal? [If relevant info discussed in review of dash, summarize here]

____
____
____
____

- <u>If goal met</u>: Good for you! Do you think that this could have any impact on [summarize hopes]?

____
____
____
____

- <u>If goal not met</u>: Okay. Let's see what we can learn about this situation. What is your sense of things that got in the way?

____
____
____
____

3. Goal Setting

Let's think about your goals for using *LiveWell* in the week ahead.

[Use FLOWSHEET to establish appropriate goals.]

- If recommending a change in goal for participant:

[If recommending change because of (1) higher priority issue arose (symptoms, targets, tasks), (2) repeated failure, or (3) entering maintenance, then provide explanation.]

It sounds like [summarize relevant target or symptoms]. It seems like [target or app use] makes the most sense to focus on for next week. How about trying to [new goal]. How does that sound?

____

____

____

- If not recommending a change in goal for participant:

[If not recommending change, let the participant decide what they want to do. If they have failed a goal one week, encourage them to try again. If they are in maintenance and have had the same goal for a while, suggest they try something new.]

It sounds like [summarize performance on goal]. Can you think of a goal that's related to the application or staying well?

[If struggling to come up with goal, suggest something.]

____

____

____

____

How do you think [above goal] might help you?

____

____

____

____

Is there anything that might make it difficult to do this?

____
____
____
____

What might help you overcome this obstacle?

____
____
____
____

4. <u>Lessons and Toolbox</u>

Now we can briefly review the lessons. It looks like [you did get a chance to read them/you didn't get a chance to read them].

[If they didn't read, ask what may have gotten in the way then skip to Toolbox section. Say they can review the lessons after the call if they'd like and follow-up with any questions they have].

The <u>Overview</u> of the *LiveWell* Program focused on how self-management along with medications can help you stay well; the <u>Basic Facts</u> focused on symptoms, early warning signs, treatment options, and getting support and information about bipolar disorder.

What are your thoughts and feelings about the material?

____
____
____
____

Do you have any questions about the information presented?

____
____
____
____

<u>Toolbox</u>
You will become increasingly aware that different parts of the application point you to skills in the Toolbox. Have you had a chance to look at the Toolbox?

____
____
____
____

Study ID:__________ Coach:__________ Date of session:__________

If you want to navigate there now with me, you'll see there are 5 categories:

- Making Changes: get motivated, make goals, follow through
- Self-Assessment: targets awareness and action/preparedness
- Lifestyle: reduce risk with SMARTS (skills for a healthy lifestyle)
- Coping: depression – dial up, mania – dial down
- Team: prepare and plan with psychiatrist, supports, hospital

How do you think the Toolbox might be helpful?

____
____
____
____

There is no explicit schedule for using the assessments or learning and practicing skills that are in the Toolbox; they are available for you to peruse on your own when you feel like you want or need to.

If you find a skill you like, you can choose to add it to your "My Skills" section of the wellness plan. Do you want to try adding a skill together now?

[If confusion or they want to add a skill, let them choose any skill and instruct how to add. If they don't want to, move on].

5. Close

Sounds like you have a great plan for the upcoming week. You are going to try to [summarize goal].

Also read about Medications and Lifestyle Skills in Foundations before we talk next week.

Can we set a time for next week? ______________________

If anything comes up before then, please feel free to call me. Thanks for talking with me today.

LiveWell: Scheduled Coaching Calls Pilot Study FV

Study ID:__________ Coach:__________ Date of session:__________

WEEK 2

Get prepared with data since last call…

Medications:	Sleep:	Routine:	Wellness Ratings:	Use of App (check ins):
Weekly Goals (week 1):				
Hopes (Initial session):				

Coach only:

Daily Review feedback category:	LiveWell Clinical Status:	Weekly Survey Scores	
		PHQ8	ASRM

1. **<u>Aims</u>**

Hi [participant]. This is your coach, [name], from the *LiveWell* program. I'm calling for our Week 2 check in. It should take about 15 minutes. Is that alright?

Today, we are scheduled to review 2 lessons (Medications and Lifestyle Skills), how using the daily check in and daily review went, and your goals from last week. We will also become more familiar with the Wellness Plan.

Is there anything else you would like to cover?

____
____
____

Any problems, questions, or concerns about using the application?

____
____
____
____

Study ID:__________ Coach:__________ Date of session:__________

2. <u>Review</u>

Let's talk about how things are going. Based on the data I have received, it looks like… [summarize dashboard data including wellness ratings, symptoms, targets, use of application].

Does that sound right?

___
___
___
___

[Support awareness, action, and success with targets. Explore problems with targets, ews monitoring, and implementing plan.]

[Only revise targets after repeated failures unless good reason to do so (i.e. started new job and schedule has shifted).]

<u>Goals</u>

Last time you hoped [week 1 goal]. To what extent were you able to achieve this goal?
[If relevant info discussed in review of dash, summarize here]

___
___
___
___

- <u>If goal met</u>: Good for you! Do you think that this could have any impact on [summarize hopes]?

___
___
___
___

- <u>If goal not met</u>: Okay. Let's see what we can learn about this situation. What is your sense of things that got in the way?

___
___
___
___

3. Goal Setting

Let's think about your goals for using *LiveWell* in the week ahead.

[Use FLOWSHEET to establish appropriate goals.]

- If recommending a change in goal for participant:

[If recommending change because of (1) higher priority issue arose (symptoms, targets, tasks), (2) repeated failure, or (3) entering maintenance, then provide explanation.]

It sounds like [summarize relevant target or symptoms]. It seems like [target or app use] makes the most sense to focus on for next week. How about trying to [new goal]. How does that sound?

____
____
____

- If not recommending a change in goal for participant:

[If not recommending change, let the participant decide what they want to do. If they have failed a goal one week, encourage them to try again. If they are in maintenance and have had the same goal for a while, suggest they try something new.]

It sounds like [summarize performance on goal]. Can you think of a goal that's related to the application or staying well?

[If struggling to come up with goal, suggest something.]

____
____
____
____

How do you think [above goal] might help you?

____
____
____
____

Is there anything that might make it difficult to do this?

____
____
____
____

What might help you overcome this obstacle?

____
____
____
____

4. **Lessons and Wellness Plan**

Now we can briefly review the lessons. It looks like [you did get a chance to read them/you didn't get a chance to read them].

[If they didn't read, ask what may have gotten in the way then skip to Wellness Plan section. Say they can review the lessons after the call if they'd like and follow-up with any questions they have].

The Medications provided basic information about medications and how to overcome common barriers to using medications effectively; and the Lifestyle Skills focused on how to promote wellness through sleep, medications, attending, routine, tranquility, and socialization.

What are your thoughts and feelings about the material?

____
____
____
____

Do you have any questions about the information presented?

____
____
____
____

After the phone training I updated your personalized anchors for the wellness rating scale that you are using in the Daily Check In. These anchors are in the Wellness Plan. Let's navigate there now and make sure everything looks ok.

You'll notice the Wellness Plan has 3 sections.

- My Resources:
  - My Medications – check if correct, please contact me to update if meds change
  - My Team – check that app has correct psychiatrist info
  - My Charts – reviews targets, might use to assist with monitoring targets
  - My Skills – where skills from the toolbox that you save show up
- Reduce Risk:
  - SMARTS – click on button to learn about related skills
  - Your personal goals for sleep, meds, routine from initial meeting
  - Generic plans if goals not met, will personalize in week 4 call
- Awareness & Action:
  - Review standard definitions
  - Check anchors, can update
  - Review standard plan, will personalize in week 4 call

You will learn about developing your Wellness Plan at the end of the Foundations.
We will personalize it during our call in week 4.

What do you think about the Wellness Plan? Any questions?

____
____
____
____

5. <u>Close</u>

Sounds like you have a great plan for the upcoming week. You are going to try to [summarize goal].

Also read about Coping Skills and Using a Team in foundations before we talk next week.

Can we set a time for next week? ________________________

If anything comes up before then, please feel free to call me. Thanks for talking with me today.

LiveWell: Scheduled Coaching Calls Pilot Study FV

Study ID:__________ Coach:__________ Date of session:__________

WEEK 3

Get prepared with data since last call…

Medications:	Sleep:	Routine:	Wellness Ratings:	Use of App (check ins):
Weekly Goals (week 2):				
Hopes (Initial session):				

Coach only:

Daily Review feedback category:	LiveWell Clinical Status:	Weekly Survey Scores	
		PHQ8	ASRM

1. **<u>Aims</u>**

Hi [participant]. This is your coach, [name], from the *LiveWell* program. I'm calling for our Week 3 check in. It should take about 15 minutes. Is that alright?

Today, we are scheduled to review 2 lessons (Coping Skills and Team), how using the daily check in and daily review went, and your goals from last week.

Is there anything else you would like to cover?

____

____

____

Any problems, questions, or concerns about using the application?

[If applicable: Does your personalized content look up to date (anchors, meds, psych info)]?

____

____

____

____

Study ID:__________ Coach:__________ Date of session:__________

2. Review

Let's talk about how things are going. Based on the data I have received, it looks like… [summarize dashboard data including wellness ratings, symptoms, targets, use of application].

Does that sound right?

___

___

___

___

[Support awareness, action, and success with targets. Explore problems with targets, ews monitoring, and implementing plan.]

[Only revise targets after repeated failures unless good reason to do so (i.e. started new job and schedule has shifted).]

Goals

Last time you hoped [week 2 goal]. To what extent were you able to achieve this goal?
[If relevant info discussed in review of dash, summarize here]

___

___

___

___

- If goal met: Good for you! Do you think that this could have any impact on [summarize hopes]?

___

___

___

___

- If goal not met: Okay. Let's see what we can learn about this situation. What is your sense of things that got in the way?

___

___

___

___

3. Goal Setting

Let’s think about your goals for using *LiveWell* in the week ahead.

[Use FLOWSHEET to establish appropriate goals.]

- If recommending a change in goal for participant:

[If recommending change because of (1) higher priority issue arose (symptoms, targets, tasks), (2) repeated failure, or (3) entering maintenance, then provide explanation.]

It sounds like [summarize relevant target or symptoms]. It seems like [target or app use] makes the most sense to focus on for next week. How about trying to [new goal]. How does that sound?

____
____
____

- If not recommending a change in goal for participant:

[If not recommending change, let the participant decide what they want to do. If they have failed a goal one week, encourage them to try again. If they are in maintenance and have had the same goal for a while, suggest they try something new.]

It sounds like [summarize performance on goal]. Can you think of a goal that’s related to the application or staying well?

[If struggling to come up with goal, suggest something.]

____
____
____

How do you think [above goal] might help you?

____
____
____
____

Is there anything that might make it difficult to do this?

____
____
____

Study ID:__________ Coach:__________ Date of session:__________

What might help you overcome this obstacle?

____

____

____

____

4. **Lessons**

Now we can briefly review the lessons. It looks like [you did get a chance to read them/you didn't get a chance to read them].

[If they didn't read, ask what may have gotten in the way then skip to Close. Say they can review the lessons after the call if they'd like and follow-up with any questions they have].

The Coping Skills provided basic information on how to dial up when depressed and dial down when manic; and the Using a Team Effectively focused on building supports, good rapport with your psychiatrist, and identifying a hospital should the need arise.

What are your thoughts and feelings about the material?

____

____

____

____

Do you have any questions about the information presented?

____

____

____

____

5. **Close**

Sounds like you have a great plan for the next week. You are going to try to [summarize goal].

Also read about Awareness and Action in the foundations and think about how you might want to personalize your Wellness Plan before we talk next week. Coming up with some ideas ahead of time will help our call go more smoothly.

Can we set a time for next week? The call will be a little bit longer, probably will take about 30-40 minutes. ______________________

If anything comes up before then, please feel free to call me. Thanks for talking with me today.

LiveWell: Scheduled Coaching Calls Pilot Study FV

Study ID:__________ Coach:__________ Date of session:__________

WEEK 4

Get prepared with data since last call…

Medications:	Sleep:	Routine:	Wellness Ratings:	Use of App (check ins):
Weekly Goals (week 3):				
Hopes (Initial session):				

Coach only:

Daily Review feedback category:	LiveWell Clinical Status:	Weekly Survey Scores	
		PHQ8	ASRM

1. **<u>Aims</u>**

Hi [participant]. This is your coach, [name], from the *LiveWell* program. I'm calling for our Week 4 check in. It should take about 30 or 40 minutes. Is that alright?

Today, we are scheduled to review the last two lessons on awareness and action, how using the daily check in and daily review went, and your goals from last week. We will also develop your personal Wellness Plan.

Is there anything else you would like to cover?

____
____
____

Any problems, questions, or concerns about using the application?

____
____
____
____

Study ID:__________ Coach:__________ Date of session:__________

2. Review

Let's talk about how things are going. Based on the data I have received, it looks like… [summarize dashboard data including wellness ratings, symptoms, targets, use of application].

Does that sound right?

____
____
____
____

[Support awareness, action, and success with targets. Explore problems with targets, ews monitoring, and implementing plan.]

[Only revise targets after repeated failures unless good reason to do so (i.e. started new job and schedule has shifted).]

Goals

Last time you hoped [week 3 goal]. To what extent were you able to achieve this goal?
[If relevant info discussed in review of dash, summarize here]

____
____
____
____

- If goal met: Good for you! Do you think that this could have any impact on [summarize hopes]?

____
____
____
____

- If goal not met: Okay. Let's see what we can learn about this situation. What is your sense of things that got in the way?

____
____
____
____

3. Lessons

Now we can briefly review the lessons. It looks like [you did get a chance to read them/you didn't get a chance to read them].

[If they didn't read, ask what may have gotten in the way then skip to Wellness Plan section. Say they can review the lessons after the call if they'd like and follow-up with any questions they have].

The Awareness section focused on learning about your personal early warning signs and symptoms of depression and mania. The Action section offered guidance about how to manage mild, moderate, and severe symptoms.

What are your thoughts and feelings about the material?

____
____
____
____

Do you have any questions about the information presented?

____
____
____
____

4. Wellness Plan

Okay. Let's talk about your personal Wellness Plan.

We'll come up with your plan and I'll enter it into the application within the next 24 hours. Go ahead and get your study phone out or put me on speaker if you're talking on the study phone, and open up the application and go to the Wellness Plan section.

My Resources

First, let's take a look at My Resources. You'll see four buttons:

- My Medications
- My Team
- My Skills
- My Charts

LiveWell: Scheduled Coaching Calls Pilot Study FV

Study ID:__________ Coach:__________ Date of session:__________

My Skills will populate automatically for you, whenever you read about a skill in the Toolbox and select to save it into your Wellness Plan. My Charts will also automatically populate for you, based on data from your daily check-ins.

Take a look at Medications. Are they all correct? [Record changes to make]

Medication	Dosage	Frequency

Take a look at your Team. Right now it should have your psychiatrist's name and phone number. Is this information correct? Who else will be a support for you? Anyone you'd like to add?

Role	Name	Number
Psychiatrist		
Therapist		
Pharmacy		
Hospital		
Family		
Friends		
Other		

Study ID:__________ Coach:__________ Date of session:__________

Reduce Risk

Next, let's take a look at Reduce Risk.

Perhaps you remember the acronym SMARTS that we use to remember the life areas that are important for wellness: sleep, medicine, attend, routine, tranquil, and social.

Right now, you see some sample ideas for living a healthy lifestyle.

Let's replace sample ideas with your own. Pick one, two, or three things at most for each area. What sorts of things do you want to do to reduce your risk for symptoms?

Area	Goal
Sleep	
Medicine	
Attend	
Routine	
Tranquil	
Social	

LiveWell: Scheduled Coaching Calls Pilot Study FV

Study ID:__________ Coach:__________ Date of session:__________

Awareness and Action

Now let's take a look at awareness and action. First take a look at your anchors. You came up with those during the application training when you came in and met with me.

Based on what you have learned about bipolar disorder, and about yourself, you may want to make changes. Is there anything you'd like to add, remove, or re-word?

Wellness	Anchors
+4 Severe Up	
+3 Moderate Up	
+2 Mild Up	
+1 Slight Up	
0 Well	
-1 Slight Down	
-2 Mild Down	
-3 Moderate Down	
-4 Severe Down	

LiveWell: Scheduled Coaching Calls Pilot Study FV

Study ID:__________ Coach:__________ Date of session:__________

Now take a look at Plan. There are some generic suggestions in there right now. Let's change these to suit you. Pick one, two, or three things you might do when…

Wellness	Action
+4<br>Severe Up	
+3<br>Moderate Up	
+2<br>Mild Up	
+1<br>Slight Up	
0<br>Well	
-1<br>Slight Down	
-2<br>Mild Down	
-3<br>Moderate Down	
-4<br>Severe Down	

5. **Close**

Okay. So from this point forward, we recommend you review and use your Wellness Plan on a regular basis as your goal. The Daily Check Ins and Daily Reviews will help you do this. But be sure you're on track knowing how you want to Reduce Risk when you are doing well and Take Action whenever you have symptoms.

How do you think this might help you?

____

____

____

____

Is there anything that might make it difficult to do this?

____

____

____

____

What might help you overcome this obstacle?

____

____

____

____

I will update your application with the information we discussed today. Please let me know if anything looks incorrect.

Okay, so we will talk in two weeks. Can we set a time now? ________________________

If anything comes up before then, please feel free to call me. Thanks for talking with me today.

LiveWell: Scheduled Coaching Calls Pilot Study FV

Study ID:__________ Coach:__________ Date of session:__________

WEEK 6

Get prepared with data since last call…

Medications:	Sleep:	Routine:	Wellness Ratings:	Use of App (check ins):
Weekly Goals (week 4):<br>-Review and use Wellness Plan on a daily basis				
Hopes (Initial session):				

Coach only:

Daily Review feedback category:	LiveWell Clinical Status:	Weekly Survey Scores	
		PHQ8	ASRM

1. **<u>Aims</u>**

Hi [participant]. How are you doing? This is your coach, [name], from the *LiveWell* program. I'm calling for our Week 6 check in. It should take about 15 minutes. Is that alright?

Today, we are scheduled to review your wellness plan, how using the daily check in and daily review went, and your goals from the last time we talked.

Is there anything else you would like to cover?

____
____
____

Any problems, questions, or concerns about using the application? Does your Wellness Plan look up to date [plans, anchors, meds, team info]?

____
____
____
____

Study ID:__________ Coach:__________ Date of session:__________

2. Review

Let's talk about how things are going. Based on the data I have received, it looks like… [summarize dashboard data including wellness ratings, symptoms, targets, use of application].

Does that sound right?

____
____
____
____

[Support awareness, action, and success with targets. Explore problems with targets, ews monitoring, and implementing plan.]

[Only revise targets after repeated failures unless good reason to do so (i.e. started new job and schedule has shifted).]

Goals

Last time we talked about reviewing and following your Wellness Plan on a daily basis. To what extent were you able to achieve this goal?
[If relevant info discussed in review of dash, summarize here]

____
____
____
____

- If goal met: Good for you! Do you think that this could have any impact on [summarize hopes]?

____
____
____
____

- If goal not met: Okay. Let's see what we can learn about this situation. What is your sense of things that got in the way?

____
____
____
____

Study ID:__________ Coach:__________ Date of session:__________

3. Wellness Plan

Now that you've had some time with it, what do you think about your Wellness Plan?

____
____
____
____

Is there anything you want to change in your Wellness Plan?

____
____
____
____

4. Close

Okay. So from this point forward, we recommend you continue to review and use your Wellness Plan on a daily basis as your goal. The Daily Check-Ins and Daily Reviews will help you do this. But be sure you're on track knowing how you want to Reduce Risk and Take Action whenever you have symptoms.

How do you think this might help you?

____
____
____
____

Is there anything that might make it difficult to do this?

____
____
____
____

What might help you overcome this obstacle?

____
____
____
____

Study ID:__________ Coach:__________ Date of session:__________

We won't talk again until your last week of actively using the application daily, which is about 2 more weeks from now.

That last week, a 9th foundation lesson called "Wrapping Up" will be highlighted in white to remind you to read it.

Can we set a time for the next call now? ____________________________

Remember that you can always get help from me with using the application by calling or emailing. Also, if you would like to change any of your personalized content (medications, team, anchors, targets, plans), please contact me.

Also, I will be reaching out if it looks like there are problems with the watch or phone, or if it appears you are not using the application regularly.

Thanks for talking with me today.

LiveWell: Scheduled Coaching Calls Pilot Study FV

Study ID:__________ Coach:__________ Date of session:__________

Final Week (8)

Get prepared with data since last call…

Medications:	Sleep:	Routine:	Wellness Ratings:	Use of App (check ins):
Weekly Goals (week 6):<br>-Review and use Wellness Plan on a daily basis				
Hopes (Initial session):				

Coach only:

Daily Review feedback category:	LiveWell Clinical Status:	Weekly Survey Scores	
		PHQ8	ASRM

1. **<u>Aims</u>**

Hi [participant]. This is your coach, [name], from the *LiveWell* program. I'm calling for our final check in. First I'd like to congratulate you on sticking with the application for two months. How does it feel?

Today, we are scheduled to review the wrap up lesson, how using the application went, and to what extent the application helped you meet your hopes. It should take about 15 minutes.

Is there anything else you would like to cover?

____

____

____

Study ID:__________ Coach:__________ Date of session:__________

2. Review

Let's talk about how things are going. Based on the data I have received, it looks like… [summarize dashboard data including wellness ratings, symptoms, targets, use of application].

Does that sound right?

____
____
____
____

[Support awareness, action, and success with targets. Explore problems with targets, ews monitoring, and implementing plan.]

Goals

Last time we talked about reviewing and following your Wellness Plan on a daily basis. To what extent were you able to achieve this goal?
[If relevant info discussed in review of dash, summarize here]

____
____
____
____

- If goal met: Good for you! Do you think that this could have any impact on [summarize hopes]?

____
____
____
____

- If goal not met: Okay. Let's see what we can learn about this situation. What is your sense of things that got in the way?

____
____
____
____

Study ID:__________ Coach:__________ Date of session:__________

3. **Lesson**

The Wrapping Up lesson offered a summary of the program, asked some questions about how the program worked for you, and provided some questions about how you might best manage your bipolar disorder in the future.

4. **LiveWell Program**

Did the program help you reach your hopes of [summarize hopes]?

____
____
____
____

Was anything especially helpful?

____
____
____
____

What do you plan to do in the future to maintain gains and make further gains?

____
____
____
____

What do you plan to do in the future should you have any setbacks?

____
____
____
____

5. **Close**

I appreciate your taking such an active part in using the application. It has been really nice working with you.

Do you have any final concerns to discuss?

____
____
____
____

After this point, use of the application is optional. We will be in touch to schedule an appointment to return the equipment.

Thanks for talking with me today, and I wish you all the best moving forwards.

LiveWell Pilot Study: Ad Hoc Coaching Calls

Study ID:_________________ Coach: __________________ Date of session:___________

1. SEVERE SYMPTOMS (Crisis Wellness Rating)

Definition: Participant with daily wellness rating of +4 or -4.

Get prepared with data since last call…

Medications:	Sleep:	Routine:	Wellness Ratings:	Use of App (check ins):

Daily Review feedback category:	LiveWell Clinical Status:	Weekly Survey Scores	
		PHQ8	ASRM

Hi [name]. This is [coach]. How are you doing?

I'm calling to touch base. You reported having severe symptoms today. This can be a dangerous situation. I'd like us to understand what is going on. Would that be okay?

You rated yourself as a [+4/-4]. Is that right?

____
____
____
____

Remember that it is a crisis if you are engaging in dangerous behaviors, having psychotic symptoms, or are unable to maintain daily routines. This includes thoughts of hurting yourself. In any of these cases, you should call your psychiatrist or go to the nearest emergency department.

I'd like to ask a few questions to make sure you are safe right now. Is that okay?

____
____
____

- Complete suicidality protocol.

  If suicidal ideation is low or mild then complete the crisis protocol. If suicidal ideation is more severe follow the suicidal ideation protocol.

- Complete crisis protocol.

LiveWell Pilot Study: Ad Hoc Coaching Calls

Study ID:________________ Coach: ________________ Date of session:__________

2. SEVERE SYMPTOMS (Crisis elevated PHQ8 or ASRM Score)

Definition: Participant with new onset of PHQ8 ≥ 10 or new onset of ASRM ≥ 6.

Get prepared with data since last call…

Medications:	Sleep:	Routine:	Wellness Ratings:	Use of App (check ins):

Daily Review feedback category:	LiveWell Clinical Status:	Weekly Survey Scores	
		PHQ8	ASRM

Hi [name]. This is [coach]. How are you doing?

I'm calling to touch base. You reported having severe symptoms today. This can be a dangerous situation. I'd like us to understand what is going on. Would that be okay?

Your most recent weekly depression/mania rating scale was high. Does that seem right?

____
____
____
____

Remember that it is a crisis if you are engaging in dangerous behaviors, having psychotic symptoms, or are unable to maintain daily routines. This includes thoughts of hurting yourself. In any of these cases, you should call your psychiatrist or go to the nearest emergency department.

I'd like to ask a few questions to make sure you are safe right now. Is that okay?

____
____
____

- Complete suicidality protocol.

  If suicidal ideation is low or mild then complete the crisis protocol. If suicidal ideation is more severe follow the suicidal ideation protocol.

- Complete crisis protocol.

LiveWell Pilot Study: Ad Hoc Coaching Calls

Study ID:_________________ Coach: __________________ Date of session:___________

3. CLINICAL STATUS DISCREPANCY

<u>Definition</u>: Participants daily wellness ratings do not match their weekly lw clinical status rating and crisis and si protocols either not activated (ongoing elevated PHQ8 or ASRM) or completed with si absent, low or mild.

PHQ8 ≥ 10	LiveWell Clinical Status from last 7 days of Daily Check Ins not unwell
ASRM ≥ 6	LiveWell Clinical Status from last 7 days of Daily Check Ins not unwell
PHQ8 < 10	LiveWell Clinical Status from last 7 days of Daily Check Ins unwell
ASRM < 6	LiveWell Clinical Status from last 7 days of Daily Check Ins unwell

Get prepared with data since last call…

Medications:	Sleep:	Routine:	Wellness Ratings:	Use of App (check ins):

Daily Review feedback category:	LiveWell Clinical Status:	Weekly Survey Scores	
		PHQ8	ASRM

Hi [name]. This is [coach]. How are you doing?

I'm calling to touch base. It seems like there is a difference in your daily and weekly ratings of your health and wellness. I'd like us to understand what is going on. Would that be okay?

When the daily and weekly check ins are inconsistent, we need to consider making changes in the anchors. What do you think is going on that your weekly survey said you may be [depressed, manic/ok] and your daily check ins say you are [well, prodomal, recovering/unwell]?

____

____

____

____

Would changing anchors in your Wellness Rating Scale make the application easier to use?

____

____

____

____

Ok, so we will talk again at our next scheduled appointment. I appreciate your taking such an active part in our conversation today. Remember, if any difficulties arise in the future I am always happy to help out.

LiveWell Pilot Study: Ad Hoc Coaching Calls

Study ID:_________________ Coach: __________________ Date of session:___________

4. LOW DAILY CHECK-IN ADHERENCE

Definition: Participant missed daily check in ≥ 3 times over the last 7 days.

Get prepared with data since last call…

Medications:	Sleep:	Routine:	Wellness Ratings:	Use of App (check ins):

Daily Review feedback category:	LiveWell Clinical Status:	Weekly Survey Scores	
		PHQ8	ASRM

Hi [name]. This is [coach]. How are you doing?

I'm calling to touch base. It seems like you have not been checking in regularly on the *LiveWell* application. I'd like to understand what is going on, and to see if I can help. Would that be okay?

Any problems, questions, or concerns about using the application?

____
____
____
____

What might you or we do to get past these barriers so you can benefit most from *LiveWell*?

____
____
____
____

Ok, so we will talk again at our next scheduled appointment. I appreciate your taking such an active part in our conversation today. Remember, if any difficulties arise in the future I am always happy to help out.
