# Supplementary material for: A Smartphone-Based Self-management Intervention for Bipolar Disorder (LiveWell): User-Centered Development Approach
Source: JMIR Ment Health. 2021 Apr 12;8(4):e20424. doi: 10.2196/20424 (PMC8076988; doi:10.2196/20424)
Supplement: Multimedia Appendix 8 [file mental_v8i4e20424_app8.pdf]

LiveWell: Pilot Study Exit Interview

ID:___________ Date:________ Follow-Up Month: ____________ Interviewer:____________

Prepare for interview:
$, Check participant completed PDQ and Exit Questionnaire

Introduction

We would like you to share your thoughts about using the *LiveWell* system.

Although we like hearing positive comments, any information about problems is very helpful.

Please don't hesitate to tell us about things that didn't work so well or that you didn't like.

Overview

1. Tell me about your overall experience using the *LiveWell* application?

____

____

____

2. What, if anything, did you like or find useful about using the application?

____

____

____

3. What did you not like or not find useful about using the application?

____

____

____

4. If you could change anything about the *LiveWell* application, what would you change?

____

____

____

LiveWell: Pilot Study Exit Interview
ID:___________ Date:________ Follow-Up Month: ____________ Interviewer:____________

Outcomes

1. What is your strongest memory from using LiveWell?

____

____

____

2. What do think worked best for you? What, if anything, made you better?

____

____

____

3. What do think worked least well for you? What, if anything, made you worse?

____

____

____

4. What impact, if any, did using LiveWell have on your life?

____

____

____

5. Did you have a clear objective when you started using LiveWell?

What was it? Did you achieve it?

____

____

____

6. How would you describe the idea behind how LiveWell is meant to help you?

____

____

____

LiveWell: Pilot Study Exit Interview
ID:___________ Date:________ Follow-Up Month: ____________ Interviewer:____________

Targets

1. How has your participation in LiveWell affected your medication use?

____

____

____

2. How has your participation affected the duration of sleep you get each day? Your sleep habits?

____

____

____

3. How has your participation affected your daily routine?

____

____

____

4. How has your participation affected your ability to identify, monitor, and manage early warning signs? What about lingering symptoms?

____

____

____

LiveWell: Pilot Study Exit Interview

ID:___________ Date:________ Follow-Up Month: ____________ Interviewer:____________

Foundations

1. What was your overall experience with the Foundations?

____

____

____

2. What, if anything, did you like or find useful about the lessons?

____

____

____

3. What did you find confusing, unclear, or not useful about the lessons?

____

____

____

4. What did you feel was missing or that you would have liked to learn more about?

   [For each topic area covered in a lesson, should there be more details about which skills to practice?]

   [Should the lessons should be more interactive (e.g. providing tools to practice specific skills)?]

____

____

____

Toolbox

1. What was your overall experience with the Toolbox section?

____

____

____

2. Tell me about your use of the Toolbox? [When, Why, How]

____

____

____

3. What, if anything, did you like or find useful about the Toolbox?

____

____

____

4. What did you find confusing, unclear, or not useful about the Toolbox?

____

____

____

5. What would help you to select and practice skills regularly? [specific skills for each lesson, more interactive skills tools, more instructions, worksheets, selecting and discussing skills practiced with the coach]

____

____

____

6. If you could change something about the Toolbox, what would that be?

____

____

____

<u>Wellness Plan</u>

1. What was your overall experience with the Wellness Plan?

____

____

____

2. What, if anything, did you like or find useful about the Wellness Plan?

____

____

____

3. What did you find challenging, unclear, or not useful about the Wellness Plan?

____

____

____

4. If you could change anything about the Wellness Plan, what would you change?

____

____

____

Wellness Plan: My Resources

1. What did you think about the My Resources section?

   [Show on phone: My Medications, My Team, My Skills, My Charts]

____

____

____

2. Can you tell me about your use of this section? [When, Why, How]

   [Use of My Meds; Use of My Team; Use of My Skills; Review of charts]

____

____

____

3. What, if anything, did you like or find useful about My Resources?

____

____

____

4. What did you find challenging, unclear, or not useful about My Resources?

____

____

____

5. If you could change anything about My Resources, what would you change?

____

____

____

Wellness Plan: Reduce Risk

1. What did you think about the Reduce Risk section?
   [Show on phone: personalized SMARTS and links to related skills]

____

____

____

2. Can you tell me about your use of this section? [When, Why, How]
   [Sleep, Meds, Abstain, Routine, Tranquil, Social]

____

____

____

3. What, if anything, did you like or find useful about Reduce Risk?

____

____

____

4. Was there anything you found challenging, unclear, or not useful about Reduce Risk?

____

____

____

5. If you could change anything about Reduce Risk, what would you change?

____

____

____

<u>Wellness Plan: Awareness & Action</u>

1. What did you think about the Awareness & Action section?

   [Show on phone; personalized anchors and plan, definition of wellness ratings]

____

____

____

2. Can you tell me about your use of this section? [When, Why, How]

____

____

____

3. What, if anything, did you like or find useful about Awareness & Action?

____

____

____

4. Was there anything you found challenging, unclear, or not useful about Awareness & Action?

____

____

____

5. If you could change anything about Awareness & Action, what would you change?

____

____

____

LiveWell: Pilot Study Exit Interview
ID:___________ Date:________ Follow-Up Month: ____________ Interviewer:____________

Daily Check In

1. What was your overall experience with the Daily Check In?

2. Can you walk me through your typical use of the Daily Check In?

3. Did you experience any difficulties completing the Daily Check In?
   [anything you did to make completing the Daily Check In easier]

4. What, if anything, did you like or find useful about the Daily Check In?

5. Was there anything you found challenging, missing, or not useful about the Check In?

6. If you could change anything about the Daily Check In, what would you change?

LiveWell: Pilot Study Exit Interview
ID:___________ Date:________ Follow-Up Month: ____________ Interviewer:____________

Daily Review

1. What was your overall experience with the Daily Review?

____
____
____

2. What, if anything, did you like or find useful about the Daily Review?

____
____
____

3. Was there anything you found confusing, unclear, or not useful about the Daily Review?

____
____
____

4. What did you think about the feedback in the Daily Review?

____
____
____

5. How did you use it? Did you follow up on any of the feedback? Did you make any changes?

____
____
____

6. If you could change anything about the Daily Review, what would you change?

____
____
____

LiveWell: Pilot Study Exit Interview
ID:___________ Date:________ Follow-Up Month: ____________ Interviewer:____________

Weekly Surveys

1. Overall, how would you describe your experience completing the Weekly Surveys?

___

___

___

2. What, if anything, did you like or find useful about completing the Weekly Surveys?

___

___

___

3. Was there anything you found challenging, unclear, or not useful regarding the Weekly Surveys or its specific questions?

___

___

___

4. What would you change about the Weekly Surveys? [Explore thoughts on ews checklists]

___

___

___

LiveWell: Pilot Study Exit Interview
ID:___________ Date:________ Follow-Up Month: ____________ Interviewer:____________

Reminders

1. What was your overall experience with the daily reminders? [Reliability – received, on time]

____
____
____

2. Were the daily reminders useful? How so?

____
____
____
____

3. Was there anything about the daily reminders you didn't like?

____
____
____

4. How do you think the reminders could be improved?

____
____
____

LiveWell: Pilot Study Exit Interview

ID:___________ Date:________ Follow-Up Month: ____________ Interviewer:____________

Clinical Status Summary

1. Did you look at the summary reports? How often? If not, why not?

____

____

____

2. Any questions, thoughts, or feedback about the report?

____

____

____

3. Are there any additional items that would have been useful to have in the report?

____

____

____

4. Are there any items you think should be dropped from the report?

____

____

____

LiveWell: Pilot Study Exit Interview
ID:___________ Date:________ Follow-Up Month: ____________ Interviewer:____________

Coach

1. How would you describe your overall experience working with the coach?

2. What, if anything, did you like or find useful about working with the coach?

3. Was there anything you found difficult or not useful about working with the coach?

4. If you could change anything about the coach's involvement, what would it be?

Psychiatrist

1. In what ways, if any, has your participation in *LiveWell* affected how you work with your psychiatrist?

____

____

____

2. What changes would you suggest to the *LiveWell* program to make it more useful in terms of terms of working with your psychiatrist?

____

____

____

LiveWell: Pilot Study Exit Interview
ID:___________ Date:________ Follow-Up Month: ____________ Interviewer:____________

Use of Study Equipment

1. Can you walk me through your typical use of the study phone and watch?

   [Where did you keep it, how frequently did you check it, etc.?]

____

____

____

2. What difficulties, if any, did you have using the study phone every day?

____

____

____

3. What difficulties, if any, did you have using the watch all day every day?

____

____

____

4. Anything you wish you'd had during the study to make it easier?

   [extra phone chargers, portable chargers, car chargers, etc]

____

____

____

<u>Technical Issues</u>

1. What technical issues, if any, did you experience with the phone or watch? [trouble with reception, battery life, receiving and responding to prompts, completing daily check-ins and/or weekly surveys]

____

____

____

If issues present, did you feel like staff was responsive in helping you address these issues?

____

____

____

If issues present, did these technical issues affect your intended use of the application? How?

____

____

____

<u>Privacy</u>

1. What concerns, if any, did you have about your privacy while using the system?

____

____

____

Thank you for your valuable feedback, is there anything else you'd like to add before we end?

____

____

____

____

____

LiveWell: Pilot Study Exit Interview
ID:___________ Date:________ Follow-Up Month: ____________ Interviewer:____________

[Administer PDQ Exit Questionnaire if not submitted prior to interview]

[Collect study materials]

Interviewer Comments
